# Subfecundity, Infertility Treatment, and Child Neurodevelopment

**DOI:** 10.1001/jamanetworkopen.2026.17324

**Published:** 2026-06-08

**Authors:** Linda G. Kahn, Alison E. Hipwell, Joseph B. Stanford, Noya Galai, Haozuo Zhao, Akram N. Alshawabkeh, Judy L. Aschner, Emily S. Barrett, Ricardo P. Bertolla, Kim Nail Cajachagua Torres, Carlos A. Camargo, Jose F. Cordero, Lisa A. Croen, Sean C. Deoni, Semsa Gogcu, Julie B. Herbstman, Margaret R. Karagas, Kaja Z. LeWinn, Kristen Lyall, Cynthia T. McEvoy, Kimberlee McKay, Thomas G. O’Connor, J. Richard Pilsner, Susan L. Schantz, Rebecca J. Schmidt, Alicia K. Smith, Greta N. Wilkening, E. Zhang, Yeyi Zhu, Akhgar Ghassabian

**Affiliations:** 1 New York University Grossman School of Medicine, New York, New York; 2 University of Pittsburgh, Pittsburgh, Pennsylvania; 3 University of Utah, Salt Lake City; 4 Johns Hopkins University, Baltimore, Maryland; 5 Northeastern University, Boston, Massachusetts; 6 Hackensack Meridian School of Medicine, Nutley, New Jersey; 7 Rutgers School of Public Health, Piscataway, New Jersey; 8 Wayne State University School of Medicine, Detroit, Michigan; 9 Harvard University Medical School, Boston, Massachusetts; 10 University of Georgia, Athens; 11 Kaiser Permanente Northern California Division of Research, Pleasanton; 12 Bill and Melinda Gates Foundation, Seattle, Washington; 13 Atrium Health Wake Forest Baptist, Winston-Salem, North Carolina; 14 Columbia University Mailman School of Public Health, New York, New York; 15 Dartmouth University Geisel School of Medicine, Hanover, New Hampshire; 16 University of California, San Francisco, San Francisco; 17 Drexel University, Philadelphia, Pennsylvania; 18 Oregon Health and Science University, Portland; 19 Avera Research Institute, Sioux Falls, South Dakota; 20 University of Rochester Medical Center, Rochester, New York; 21 University of Illinois at Urbana-Champaign, Champaign; 22 University of California, Davis; 23 Emory University School of Medicine, Atlanta, Georgia; 24 University of Colorado School of Medicine, Aurora; 25 University of Kansas Medical Center, Kansas City

## Abstract

**Importance:**

Increasing numbers of children are conceived using infertility treatment; concerns remain about potential effects on child neurodevelopment.

**Objective:**

To evaluate whether infertility treatment is associated with child neurodevelopment and whether such an association may be attributable to underlying subfecundity.

**Design, Setting, and Participants:**

This cohort study was conducted among mother-child dyads in the National Institutes of Health Environmental Influences on Child Health Outcomes Cohort, with infants conceived between 1998 and 2022. Associations of subfecundity and infertility treatment with neurodevelopmental outcomes were assessed among children ages 2 to 10 years. Data were analyzed from May 14, 2025, to March 31, 2026.

**Exposure:**

Subfecundity was defined as prior consultation for, treatment of, or diagnosis of infertility for either partner; at least 2 prior miscarriages; or ever having had unprotected heterosexual intercourse for 12 months without conceiving. Infertility treatment was categorized as in vitro fertilization (IVF) or non-IVF treatment.

**Main Outcomes and Measures:**

Harmonized caregiver responses to the Strengths and Difficulties Questionnaire and the Child Behavior Checklist yielded continuous raw scores for externalizing and internalizing problems. The total raw Social Responsiveness Scale (SRS) score quantified autism-like symptoms. Caregivers reported physician diagnosis of autism spectrum disorder (ASD) and attention deficit/hyperactivity disorder (ADHD).

**Results:**

Among 15 382 mother-infant dyads, there were 14 191 unique maternal participants (mean [SD] age at delivery, 30.9 [5.33] years; 8780 parous participants [57.1%]). ASD and ADHD were diagnosed in 876 offspring (7.6%) and 819 offspring (7.1%), respectively. In generalized linear models, subfecundity was associated with higher externalizing problem and SRS scores among all pregnancies (externalizing problems: *b* = 0.47 [95% CI, 0.14-0.81]; SRS score: *b* = 1.08 [95% CI, 0.01-2.14]) and when restricted to natural conceptions (externalizing problems: *b* = 0.45 [95% CI, 0.07-0.83]; SRS score: *b* = 1.12 [95% CI, −0.09 to 2.34]). Offspring of parents with subfecundity had higher odds of ASD (overall: odds ratio [OR], 1.27 [95% CI, 1.03-1.57]; natural conceptions: OR, 1.31 [95% CI, 1.04-1.64]). Children conceived via non-IVF treatment had higher odds of ADHD compared with those conceived via natural conception with subfecundity (OR, 1.77 [95% CI, 1.16-2.68]) or without subfecundity (OR, 1.54 [95% CI, 1.05-2.25]). There were no significant associations for IVF treatment.

**Conclusions and Relevance:**

In this large US cohort study, subfecundity was associated with elevated scores for caregiver-reported symptoms of behavioral problems and higher odds of ASD diagnosis, independent of infertility treatment. Non-IVF treatment was associated with ADHD, warranting further research into specific indications for treatment that may increase risk of offspring neurodevelopmental problems.

## Key Points

**Question:**

Is infertility treatment or underlying subfecundity associated with adverse child neurodevelopment?

**Findings:**

In this cohort study including a national sample of 15 382 mother-infant dyads, subfecundity was associated with more behavior problems, more autism-like symptoms, and higher odds of autism spectrum disorder among offspring, even among those who were conceived naturally. In a subsample with infertility treatment data, non-IVF infertility treatment was associated with higher odds of offspring attention-deficit/hyperactivity disorder compared with natural conception with or without subfecundity.

**Meaning:**

This cohort study found that subfecundity was associated with adverse child neurodevelopment independent of infertility treatment; specific indications for non-IVF infertility treatment may explain part of the observed association with attention-deficit/hyperactivity disorder.

## Teaser

This cohort study evaluates whether infertility treatment is associated with child neurodevelopment and whether such associations may be attributable to underlying subfecundity.

## Introduction

Since the advent of assisted reproduction, concerns have been raised about potential negative effects on child neurodevelopment. There is some evidence that exogenous hormones prescribed during in vitro fertilization (IVF) and other assisted reproduction protocols may influence endogenous hormone production in a way that impairs offspring brain development.^1^ Even among singletons, IVF is associated with elevated rates of preterm birth and low birth weight,^2^ which in turn have adverse neurodevelopmental sequelae.^3^ More than 3 dozen studies have examined potential links between assisted reproduction and offspring neurodevelopment, most of which found no associations with cognitive, behavioral, motor, or language outcomes; autism spectrum disorder (ASD); or mental health problems.^4^ However, with the exception of several large record-linkage studies, most of these analyses have been small or of moderate size (<2500 participants), have had limited follow-up, and have not examined the role of underlying subfecundity—a potential source of confounding by indication.

The few studies that have considered subfecundity separately from infertility treatment suggest that underlying subfecundity, not IVF, may be responsible for any increased risk to offspring neurodevelopment. For example, Diop and colleagues^5^ used pregnancy and early intervention (EI) records and national IVF reporting data to examine the prevalence of EI enrollment in Massachusetts among singleton children born to mothers who conceived via IVF, to mothers with a history of subfecundity who conceived naturally, and to mothers with no history of subfecundity. Results showed that the odds of EI enrollment between ages 0 and 3 years were similarly elevated among both the IVF and subfecundity groups compared with the fecund group. Because Diop et al^5^ did not stratify by type of developmental delay, and children with both risk of and established developmental delays of any kind are eligible for EI in Massachusetts, it cannot be concluded that their finding was specifically associated with neurodevelopment. Using the same approach in a later study, Diop et al^6^ reported that neither IVF nor subfecundity was associated with ASD in the offspring. Another record-linkage study in Canada found that children whose parents had a history of infertility but did not use infertility treatment, children conceived via non-IVF infertility treatment, and children conceived via IVF had similarly elevated rates of attention-deficit/hyperactivity disorder (ADHD) relative to naturally conceived children whose parents had no history of infertility.^7^ Due to their reliance on registry data, these studies were limited in their ability to refine their subfecundity measure or examine subclinical neurodevelopmental outcomes.

In this study, we sought to disentangle the association of infertility treatment with adverse child neurodevelopmental outcomes from that of underlying subfecundity using data from a large US cohort. We extended prior research by comparing caregiver-reported behavioral problems, autism-like symptoms, and neurodevelopmental diagnoses among children born to those with subfecundity vs no subfecundity, and also contrasted outcomes among those who conceived using IVF or non-IVF treatment with those who conceived naturally, with and without subfecundity.

## Methods

### Study Population

This cohort study uses data from the National Institutes of Health Environmental Influences on Child Health Outcomes (ECHO) Cohort, a longitudinal prospective study of cohort sites across the US, including Puerto Rico, that collect data and biospecimens throughout a child’s life course from pregnancy through age 20 years with the goal of studying environmental factors associated with child health.^8^ Individual protocols were approved by the institutional review boards at each ECHO site. Written informed consent was obtained from all adult participants. This study is reported following the Strengthening the Reporting of Observational Studies in Epidemiology (STROBE) reporting guideline.

Our analysis included 15 382 mother-child dyads from 44 ECHO sites that had collected data on at least 1 fecundity exposure measure and 1 child neurodevelopmental outcome between ages 2 and 10 years by October 31, 2023. Pregnancies were conceived between 1998 and 2022. We included 1 randomly selected child from multiple gestations. We excluded adoption cohorts, pregnancies conceived with donor gametes, and observations from sites with relevant data from fewer than 5 pregnancies (Figure).

**Figure.**
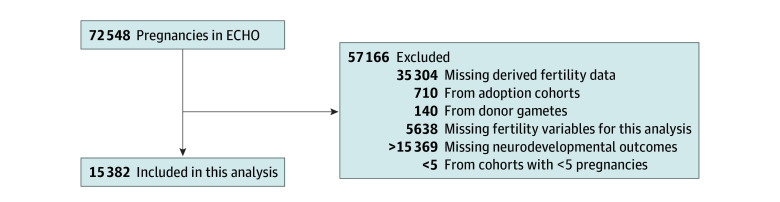
Flowchart of Sequential Exclusions to Create the Final Analytic Dataset

### Exposures

Sites collected data on reproductive history and means of conception using their own surveys supplemented by information from participants’ medical records. These data were harmonized to create an overall subfecundity measure, defined as prior consultation for, treatment of, or diagnosis of infertility for either partner; at least 2 known prior miscarriages; or ever having had unprotected heterosexual intercourse for 12 months without conceiving. In addition, IVF treatment or non-IVF treatment for the pregnancy resulting in the index child’s birth were identified as separate variables. Available IVF treatment data did not distinguish between subtypes (eg, fresh vs frozen embryo transfer, use of intracytoplasmic sperm injection). Non-IVF treatments included ovulation induction medication, intrauterine insemination, or other treatments.

### Outcomes

Sites administered the Strengths and Difficulties Questionnaire (SDQ),^9^ the Child Behavior Checklist (CBCL),^10^ and/or the Social Responsiveness Scale–Second Edition (SRS-2)^11^ to caregivers to assess neurodevelopmental outcomes in early and mid-childhood. Results from the SDQ and CBCL were harmonized to create continuous scores for externalizing problems (eg, aggression) and internalizing problems (eg, anxiety), with ranges of 0 to 72.5 and −0.2 to 70.5, respectively, with higher scores indicating greater problems.^12^ Autism-like symptoms were quantified via the total SRS score (minimum = 0; maximum = 195), with higher scores indicating more autism-like symptoms. For all continuous outcomes, we used raw scores and adjusted for child age and sex in our models.^10^ Medical records and caregiver report of physician diagnosis were used to determine child ADHD and ASD status. Neurodevelopment was assessed at least once during either the preschool-age (age 2-5 years) or school-age (age 6-10 years) years. If a child was assessed during both intervals, we used the results of the earlier assessment, as it was more proximal to the time of exposure.

### Covariates

We considered a number of covariates as potential confounders based on a directed acyclic graph (eFigure in Supplement 1) or as precision variables informed by literature supporting their association with child neurodevelopment: maternal age, self-reported race (Asian, Black, White, multiple races, and other [American Indian or Alaska Native, Hawaiian or Pacific Islander, and other nonspecified]) and ethnicity (Hispanic or non-Hispanic), prepregnancy body mass index (BMI; calculated as weight in kilograms divided by height in meters squared), education, health insurance coverage, parity, prenatal tobacco use, prenatal alcohol use, prenatal stress, and prior diagnosis of a psychiatric disorder; household income; and child sex and age at SDQ, CBCL, or SRS assessment. Health insurance type and perceived stress score^13^ during pregnancy were initially considered as potential covariates but were not included in regression analyses, as more than 50% of participants were missing data on these variables. We also chose not to include paternal age, as it had relatively high missingness (30%) and was strongly correlated with maternal age (*r* = 0.71; *P* < .001). Child birth weight, gestational age at delivery, delivery mode, and plurality were not adjusted for, as they were considered potential mediators.

### Statistical Analysis

We calculated descriptive statistics for characteristics of the total sample and stratified by exposure status. We explored correlations among the 3 continuous outcomes (externalizing, internalizing, and SRS scores) to determine whether or not to model the outcomes jointly.

Multiple imputation by chained equations was applied for covariates missing less than 25% in the total sample, with 30 iterations per imputed dataset, for 20 imputed datasets.^14^ To account for differences in cohort characteristics, imputation was conducted within 5 subgroups defined by cohort type: 32 general population cohorts; 4 autism risk–enriched, 5 preterm birth– or neonatal intensive care unit–enriched, and 1 asthma-enriched cohorts; and 2 randomized clinical trials involving pregnant smokers.

To examine associations of exposures with neurodevelopmental outcomes, we applied a generalized linear model framework with nested random effects for mother and cohort site to account for correlations resulting from repeated pregnancies and participants coming from the same ECHO site, respectively. Correlations of the continuous outcome variables were moderately positive between externalizing and internalizing scores (*r* = 0.63; *P* < .001), between externalizing and SRS scores (*r* = 0.49; *P* < .001), and between internalizing and SRS scores (*r* = 0.54; *P* < .001). Based on these results and the conceptual assumption of potential correlation between the externalizing and internalizing scores, these 2 outcomes were modeled jointly, and SRS was modeled separately. Use of a multivariate (multitarget) model for correlated outcomes compared with a series of independent univariate (single-target) models has the advantages of improved precision and the ability to account for the potential correlation of the effect of the exposure with the outcomes, while simultaneously accounting for the issue of multiple comparisons.^15,16^ The continuous outcomes were modeled with linear mixed-effects regression while the binary outcomes of ASD and ADHD diagnoses were modeled with logistic mixed-effects regression.

We fitted bivariate models with each exposure-outcome combination to assess unadjusted associations, followed by multivariable models adjusted for the aforementioned confounders and precision variables, with additional adjustment for child age in models with continuous outcomes. We also tested for interaction by child sex and child age group (preschool age vs school age) at assessment.

To assess the stability of the results observed in the full sample, we performed sensitivity analyses restricted to participants with complete data available and to cohorts recruited from the general population. We also examined the contrast between participants who conceived naturally and had subfertility vs no subfertility among the subset of ECHO sites that collected information on infertility treatment type. In a post hoc analysis, we conducted mediation analysis to examine potential mediation of significant findings from our main models by gestational age at birth and examined the prevalence of diagnosis of polycystic ovarian syndrome (PCOS) among participants in the various exposure groups.

*P* values were 2-sided, and statistical significance was set at *P* ≤ .05. Data were analyzed using R software version 4.5.0 (R Project for Statistical Computing) from May 14, 2025, to March 31, 2026.

## Results

Fecundity data were available for 15 382 pregnancies, representing 14 191 unique maternal participants (mean [SD] age at delivery, 30.9 [5.33] years; 8780 parous participants [57.1%]). By race and ethnicity, 1046 mothers (7.4%) identified as Asian, 1428 mothers (10.1%) as Black, 9000 mothers (63.4%) as White, 694 mothers (4.9%) as multiple races, and 1037 mothers (7.3%) as other race; 3356 mothers (23.6%) identified as Hispanic and 10 636 mothers (74.9%) as non-Hispanic. Most maternal participants reported at least a high school education (14 079 participants [91.5%]), nearly half reported annual household income more than $75 000 (7054 participants [45.9%]), one-quarter had BMI of 30 or higher (3654 participants [23.8%]), and few reported alcohol or tobacco use during pregnancy (2113 participants [13.7%] and 961 participants [6.2%], respectively). One-third of maternal participants (4904 participants [34.6%]) reported ever being diagnosed with a psychiatric disorder. Participants who used IVF or other infertility treatment were more likely to be older, nulliparous, and nonsmoking; self-identify as White and non-Hispanic; and have higher household income and education level compared with those who conceived naturally (Table 1).

**Table 1.** Description of the Sample Overall and Stratified by Exposure Status

Maternal, child, and pregnancy factors	Participants, No. (%)				
	Total (N = 15 382)^a^	Natural conception		Fertility treatment	
		No subfecundity (n = 12 188)	Subfecundity (n = 2333)	Non-IVF treatment (n = 475)	IVF (n = 345)
**Maternal characteristics**					
Age at delivery, y					
Mean (SD)	30.9 (5.33)	30.6 (5.27)	31.7 (5.42)	32.4 (4.33)	36.1 (4.27)
Median (range)	31.0 (13.0-50.0)	31.0 (13.0-49.0)	32.0 (18.0-47.0)	32.0 (22.0-43.0)	36.0 (26.0-50.0)
Missing	196 (1.3)	167 (1.4)	22 (0.9)	1 (0.2)	1 (0.3)
Race^b^^,^^c^					
Asian	1046 (7.4)	877 (7.7)	94 (4.5)	27 (6.2)	40 (12.5)
Black	1428 (10.1)	1206 (10.6)	201 (9.7)	9 (2.1)	11 (3.4)
White	9000 (63.4)	7019 (62.0)	1363 (65.8)	357 (82.3)	238 (74.6)
Multiple race	694 (4.9)	532 (4.7)	126 (6.1)	20 (4.6)	14 (4.4)
Other race	1037 (7.3)	848 (7.5)	172 (8.3)	7 (1.6)	8 (2.5)
Missing	986 (6.9)	847 (7.5)	116 (5.6)	14 (3.2)	8 (2.5)
Ethnicity^b^					
Non-Hispanic	10 636 (74.9)	8362 (73.8)	1557 (75.1)	389 (89.6)	294 (92.2)
Hispanic	3356 (23.6)	2801 (24.7)	494 (23.8)	36 (8.3)	22 (6.9)
Missing	199 (1.4)	166 (1.5)	21 (1.0)	9 (2.1)	3 (0.9)
Education					
High school, GED, or equivalent	14 079 (91.5)	11074 (90.9)	2165 (92.8)	465 (97.9)	335 (97.1)
<High school	817 (5.3)	678 (5.6)	135 (5.8)	<5 (<1.1)	<5 (<1.4)
Missing	486 (3.2)	436 (3.6)	33 (1.4)	<10 (<2.1)	<10 (<2.9)
Household income, $					
<30 000	2077 (13.5)	1690 (13.9)	366 (15.7)	17 (3.6)	<5 (<1.4)
30 000-49 999	1201 (7.8)	925 (7.6)	238 (10.2)	30 (6.3)	7 (2.0)
50 000-74 999	1542 (10.0)	1196 (9.8)	290 (12.4)	38 (8.0)	17 (4.9)
≥75 000	7054 (45.9)	5516 (45.3)	935 (40.1)	310 (65.3)	271 (78.6)
Missing	3508 (22.8)	2861 (23.5)	504 (21.6)	80 (16.8)	<50 (<14.5)
Any insurance					
No	807 (5.2)	462 (3.8)	210 (9.0)	86 (18.1)	49 (14.2)
Yes	6526 (42.4)	5203 (42.7)	979 (42.0)	199 (41.9)	130 (37.7)
Missing	8049 (52.3)	6523 (53.5)	1144 (49.0)	190 (40.0)	166 (48.1)
Parity					
Nulliparous	5618 (36.5)	4513 (37.0)	670 (28.7)	239 (50.3)	187 (54.2)
Parous	8780 (57.1)	6787 (55.7)	1610 (69.0)	210 (44.2)	142 (41.2)
Missing	986 (6.4)	888 (7.3)	53 (2.3)	26 (5.5)	16 (4.6)
Prepregnancy BMI					
<18.5	370 (2.4)	290 (2.4)	69 (3.0)	7 (1.5)	<5 (<1.4)
18.5-24.9	6367 (41.4)	5145 (42.2)	831 (35.6)	190 (40.0)	182 (52.8)
25.0-29.9	3685 (24.0)	2928 (24.0)	564 (24.2)	105 (22.1)	78 (22.6)
≥30	3654 (23.8)	2708 (22.2)	720 (30.9)	153 (32.2)	62 (18.0)
Missing	1306 (8.5)	1117 (9.2)	149 (6.4)	20 (4.2)	<23 (<6.7)
Tobacco use during pregnancy					
No	13 107 (85.2)	10 282 (84.4)	2013 (86.3)	443 (93.3)	328 (95.1)
Yes	961 (6.2)	766 (6.3)	172 (7.4)	18 (3.8)	5 (1.4)
Missing	1314 (8.5)	1140 (9.4)	148 (6.3)	14 (2.9)	12 (3.5)
Alcohol use during pregnancy					
No	10 910 (70.9)	8495 (69.7)	1753 (75.1)	369 (77.7)	268 (77.7)
Yes	2113 (13.7)	1715 (14.1)	284 (12.2)	74 (15.6)	31 (9.0)
Missing	2359 (15.3)	1978 (16.2)	296 (12.7)	32 (6.7)	46 (13.3)
History of psychiatric disorder^b^^,^^c^^,^^d^					
No	8010 (56.4)	6414 (56.6)	1121 (54.1)	242 (55.8)	215 (67.4)
Yes	4904 (34.6)	3744 (33.0)	901 (43.5)	152 (35.0)	94 (29.5)
Missing	1277 (9.0)	1171 (10.3)	50 (2.4)	40 (9.2)	10 (3.1)
Perceived Stress Scale *t*-score					
Mean (SD)	47.9 (10.0)	47.9 (10.0)	48.5 (9.91)	45.7 (9.2)	43.6 (9.3)
Median (range)	47.9 (22.4-84.9)	47.9 (22.4-84.9)	48.4 (22.4-82.0)	46.0 (22.4-69.8)	41.1 (25.8-67.0)
Missing	10 476 (68.1)	8035 (65.9)	1743 (74.7)	388 (81.7)	295 (85.5)
**Child characteristics**					
Sex					
Female	7247 (47.1)	5815 (47.7)	1015 (43.5)	226 (47.6)	164 (47.5)
Male	8135 (52.9)	6373 (52.3)	1318 (56.5)	249 (52.4)	181 (52.5)
Birth weight, g					
Mean (SD)	3230 (736)	3270 (672)	3070 (939)	3100 (854)	2900 (870)
Median (range)	3330 (360-5930)	3350 (383-5930)	3290 (360-5850)	3290 (500-4990)	3130 (360-4410)
<2500	1578 (10.3)	1051 (8.6)	372 (15.9)	72 (15.2)	76 (22.0)
≥2500	13 248 (86.1)	10 691 (87.7)	1868 (80.1)	397 (83.6)	261 (75.7)
Missing	556 (3.6)	446 (3.7)	93 (4.0)	6 (1.3)	8 (2.3)
Gestational age at birth, wk					
Mean (SD)	38.2 (3.1)	38.4 (2.7)	37.3 (4.3)	37.7 (3.7)	36.9 (4.1)
Median (range)	39.0 (22.0-43.0)	39.0 (22.0-43.0)	39.0 (22.0-43.0)	39.0 (23.0-42.0)	38.0 (23.0-42.0)
Missing	46 (0.3)	28 (0.2)	17 (0.7)	0 (0)	1 (0.3)
**Pregnancy and delivery characteristics**					
Gestational age at birth					
Term birth (≥37 wk)	13 401 (87.1)	10 861 (89.1)	1871 (80.2)	388 (81.7)	248 (71.9)
Preterm birth (<37 wk)	1944 (12.6)	1303 (10.7)	449 (19.2)	87 (18.3)	97 (28.1)
Missing	37 (0.2)	24 (0.2)	13 (0.6)	0 (0)	0 (0)
Delivery mode					
Cesarean	5179 (33.7)	3784 (31.0)	981 (42.0)	221 (46.5)	183 (53.0)
Vaginal	9599 (62.4)	7965 (65.4)	1221 (52.3)	242 (50.9)	161 (46.7)
Missing	604 (3.9)	439 (3.6)	131 (5.6)	12 (2.5)	1 (0.3)
Plurality					
Singleton	14 908 (96.9)	11 922 (97.8)	2247 (96.3)	434 (91.4)	272 (78.8)
Multiples	349 (2.3)	188 (1.5)	55 (2.4)	36 (7.6)	64 (18.6)
Missing	125 (0.8)	78 (0.6)	31 (1.3)	5 (1.1)	(2.6)

Abbreviations: BMI, body mass index (calculated as weight in kilograms divided by height in meters squared); GED, general educational development; IVF, in vitro fertilization.

^a^ Includes 41 pregnancies with no specific information on infertility treatment.

^b^ Distributions for race, ethnicity, and psychiatric disorder are shown for unique participants (N = 14 191). Distributions for all other variables are presented per pregnancy, as those factors could change for repeated pregnancies contributed by the same participant.

^c^ Other race category includes American Indian or Alaska Native, Hawaiian or Pacific Islander, and other nonspecified race.

^d^ Maternal psychiatric disorders include: major depression, dysthymia, bipolar disorder, anxiety disorder (ie, generalized anxiety disorder, specific phobia, panic disorder, obsessive-compulsive disorder, social anxiety, posttraumatic stress disorder, and anxiety not otherwise specified), attention-deficit hyperactivity disorder, eating disorder, schizophrenia, alcoholism, substance abuse, or autism spectrum disorder.

Overall, 8135 children (52.9%) were male, mean (SD) birth weight was 3230 (736) g, mean (SD) gestational age was 38.2 (3.1) weeks, 1578 children (10.3%) had low birth weight (<2500 g), and 1944 (12.6%) were born preterm (<37 weeks). Most pregnancies were singleton (14 908 pregnancies [96.9%]) and delivered vaginally (9599 pregnancies [62.4%]). Compared with infants conceived naturally and via non-IVF treatment, those conceived via IVF had lower gestational age at birth and a higher proportion of multiple gestations. Those whose parents experienced no subfecundity had the lowest prevalence of preterm birth, low birth weight, and cesarean delivery (Table 1).

On average, younger children had higher raw externalizing scores than older children; internalizing and SRS scores were comparable across age groups. There were 819 children (7.1%) diagnosed with ADHD and 876 children (7.6%) diagnosed with ASD in the full sample, which included 3 autism-enriched cohorts (Table 2). Because the largest autism-enriched cohort did not collect data on infertility treatment type, the subset of cohorts that collected data on treatment type had a lower percentage of children with ASD diagnosis (315 children [3.5%]) compared with the full cohort. This restricted sample had a lower mean SRS score (mean [SD] score, 32.0 [20.7] vs 33.0 [21.9]) and a lower percentage of children with ADHD diagnosis (569 children [6.3%]) (Table 2).

**Table 2.** Distribution of Outcomes

Outcome	Total group		Subgroup with data on treatment type		Age 2-5 y (n = 10 499)		Age 6-10 y (n = 4785)	
	No.^a^	Mean (SD)	No.^a^	Mean (SD)	No.	Mean (SD)	No.	Mean (SD)
Continuous scale outcomes (raw scores)								
Harmonized CBCL/SDQ externalizing problem score	10 995	8.4 (8.0)	8832	8.2 (8.0)	8574	9.1 (8.0)	2421	5.9 (7.8)
Harmonized CBCL/SDQ internalizing problem score	10 997	5.6 (6.0)	8834	5.5 (5.8)	8574	5.7 (6.0)	2421	5.4 (5.9)
SRS score	9717	33.0 (21.9)	7916	32.0 (20.7)	6759	32.8 (20.4)	2958	33.5 (25.1)
Binary outcomes								
Attention-deficit/hyperactivity disorder diagnosis	11 457	819 (7.1)^b^	8989	569 (6.3)^b^	NA	NA	NA	NA
Autism spectrum disorder diagnosis	11 457	876 (7.6)^b^	8989	315 (3.5)^b^	NA	NA	NA	NA

Abbreviations: CBCL, Child Behavior Checklist; NA, not available; SDQ, Strengths and Difficulties Questionnaire; SRS, Social Responsiveness Scale.

^a^ Two participants were missing information on age at CBCL/SDQ assessment.

^b^ Expressed as No. (%).

When modeled jointly with internalizing scores, externalizing scores were elevated for children born to parents with subfecundity compared with those who had no subfecundity, both among all pregnancies (*b* = 0.47 [95% CI, 0.14 to 0.81]) and among natural conception pregnancies (*b* = 0.45 [95% CI, 0.07 to 0.83]) (Table 3). In separate models, SRS scores were also elevated among children whose parents had subfecundity compared with those who had no subfecundity (overall: *b* = 1.08 [95% CI, 0.01 to 2.14]; natural conception: *b* = 1.12 [95% CI, −0.09 to 2.34]). Considering the binary outcomes, children born to parents with subfecundity were more likely to have a diagnosis of ASD compared with those born to parents without subfecundity (overall: odds ratio [OR], 1.27 [95% CI, 1.03 to 1.57]; naturally conceived: OR, 1.31 [95% CI, 1.04 to 1.64]).

**Table 3.** Associations of Subfecundity With Neurodevelopmental Outcomes

Outcome	All pregnancies, subfecundity vs no subfecundity					Natural conceptions pregnancies, subfecundity vs no subfecundity				
	No.			Estimate (95% CI)^a^	No.				Estimate (95% CI)^a^
	Total	Subfecundity	No subfecundity		Total		Subfecundity	No subfecundity	
Continuous outcomes (linear models)									
Externalizing problem score^b^	10 997	2274	8723	0.47 (0.14 to 0.81)^c^	10 358		1640	8718	0.45 (0.07 to 0.83)^c^
Internalizing problem score^b^				0.23 (−0.10 to 0.57)^c^					0.14 (−0.23 to 0.52)^c^
Social Responsiveness Scale score^b^	9717	2101	7616	1.08 (0.01 to 2.14)^c^	9118		1507	7611	1.12 (−0.09 to 2.34)^c^
Categorical outcomes (logistic models)									
Attention-deficit/hyperactivity disorder diagnosis	11 455	2429	9026	1.02 (0.84 to 1.23)^d^	10 807		1785	9022	0.91 (0.73 to 1.12)^d^
Autism spectrum disorder diagnosis	11 455	2429	9026	1.27 (1.03 to 1.57)^d^	10 807		1785	9022	1.31 (1.04 to 1.64)^d^

^a^ All models are adjusted for child sex; maternal age, race, ethnicity, education, parity, prepregnancy body mass index, tobacco use during index pregnancy, alcohol use during index pregnancy, and lifetime psychiatric diagnosis; and household income, with additional adjustment of child age for continuous outcomes.

^b^ Externalizing and internalizing problem scores are based on the harmonization of Child Behavior Checklist and Strengths and Difficulties Questionnaire data; the Social Responsiveness Scale score is the total raw score.

^c^ For linear models, estimates are mean difference in scores (*b*) between the subfecundity and no subfecundity groups with 95% CIs.

^d^ Logistic model results are shown as odds ratios with 95% CIs.

Table 4 shows the associations of IVF and non-IVF infertility treatment with neurodevelopmental outcomes among the subset of participants whose cohorts collected treatment type data. Children conceived via non-IVF treatment had higher odds of ADHD compared with pregnancies that occurred via natural conception with subfecundity (OR, 1.77 [95% CI, 1.16-2.68]) or via natural conception without subfecundity (OR, 1.54 [95% CI, 1.05-2.25]). ORs were elevated, but not statistically significantly, for associations between IVF treatment and ADHD for both comparison groups.

**Table 4.** Associations of Non-IVF Treatment and IVF Treatment With Neurodevelopmental Outcomes: All Pregnancies With Available Information on Fertility Treatment

Outcomes	Compared with natural conception without subfecundity				Compared with natural conception with subfecundity			
	Non-IVF treatment		IVF treatment		Non-IVF treatment		IVF treatment	
	No.	Estimate (95% CI)^a^	No.	Estimate (95% CI)^a^	No.	Estimate (95% CI)^a^	No.	Estimate (95% CI)^a^
Continuous outcomes (linear models)								
Externalizing problem score^b^	n = 7131; 337 vs 6794	0.41 (−0.33 to 1.16)^c^	n = 7062; 268 vs 6794	0.62 (−0.22 to 1.46)^c^	n = 1772; 337 vs 1435	0.01 (−0.78 to 0.81)^c^	n = 1703; 268 vs 1435	0.22 (−0.68 to 1.11)^c^
Internalizing problem score^b^		0.52 (−0.22 to 1.26)^c^		0.44 (−0.40 to 1.28)^c^		0.38 (−0.41 to 1.18)^c^		0.31 (−0.58 to 1.20)^c^
Social Responsiveness Scale score^b^	n = 6313; 328 vs 5985	1.71 (−0.49 to 3.91)^c^	n = 6227; 242 vs 5985	0.10 (−2.50 to 2.69)^c^	n = 1689; 328 vs 1361	0.92 (−1.44 to 3.28)^c^	n = 1603; 242 vs 1361	−0.69 (−3.44 to 2.05)^c^
Categorical outcomes (logistic models)								
Attention-deficit/ hyperactivity disorder diagnosis	n = 7255; 380 vs 6845	1.54 (1.05 to 2.25)^d^	n = 7084; 239 vs 6845	1.28 (0.71 to 2.30)^d^	n = 1905; 380 vs 1525	1.77 (1.16 to 2.68)^d^	n = 1764; 239 vs 1525	1.47 (0.80 to 2.70)^d^
Autism spectrum disorder diagnosis	n = 7255; 380 vs 6845	1.01 (0.53 to 1.90)^d^	n = 7084; 239 vs 6845	0.98 (0.42 to 2.32)^d^	n = 1905; 380 vs 1525	0.84 (0.43 to 1.63)^d^	n = 1764; 239 vs 1525	0.82 (0.34 to 1.98)^d^

^a^ All models are adjusted for child sex; maternal age, race, ethnicity, education, parity, prepregnancy body mass index, tobacco use during index pregnancy, alcohol use during index pregnancy, and lifetime psychiatric diagnosis; and household income, with additional adjustment of child age for continuous outcomes.

^b^ Externalizing and internalizing problem scores are based on the harmonization of Child Behavior Checklist and Strengths and Difficulties Questionnaire data; the Social Responsiveness Scale score is the total raw score.

^c^ For linear models, estimates are mean difference in scores (*b*) between the subfecundity and no subfecundity groups with 95% CIs.

^d^ Logistic model results are shown as odds ratios with 95% CIs.

To assess whether associations differed by child sex, we fitted the models testing associations between subfecundity and neurodevelopmental outcomes with an interaction term between child sex and the exposure variable. In all models, the interaction term was not significant; therefore, we did not proceed with stratified analysis. Similarly, we observed no significant interaction with age group; thus, we omitted that stratification as well.

Results from sensitivity analyses including only the general population cohorts were similar but slightly attenuated (eTable 1 and eTable 2 in Supplement 1). Among complete cases, we observed no associations of subfecundity with SRS score or ASD (eTable 3 and eTable 4 in Supplement 1). We also confirmed that results displayed in Table 3 were generally consistent when restricted to the subset of participants in ECHO sites that collected information on type of infertility treatment (eTable 5 in Supplement 1).

Mediation analysis indicated that associations of subfecundity with both externalizing and SRS were mediated by gestational age at birth, but there was no evidence that gestational age at birth was on the causal pathway between subfecundity and ASD (eTable 6 in Supplement 1). Among 5197 pregnancies from 28 cohorts in our study that had data on both PCOS diagnosis and use of infertility treatment, nearly one-third (31%) that involved IVF and nearly one-half (49%) that involved non-IVF infertility treatment were among participants who had PCOS, while only 11% and 3% of naturally conceived pregnancies with and without subfecundity, respectively, were among participants with PCOS.

## Discussion

In a nationwide US cohort study of mother-child dyads, we found associations of subfecundity and infertility treatment with caregiver-reported symptoms of behavioral problems. Children born to parents with a history of subfecundity, whether or not they used infertility treatment, had higher odds of ASD diagnosis than those born to parents without subfecundity, as well as higher externalizing and SRS scores, although these differences were small and likely not clinically relevant. Our results support the growing consensus that the elevated risk of adverse neurodevelopmental outcomes among children conceived via infertility treatment derives from underlying pathology, not the treatment itself. Indeed, a systematic review of the literature on child neurodevelopmental outcomes following use of assisted reproduction vs natural conception^4^ found that observed associations were generally attributable to confounding by indication or by multiple gestation and preterm birth, which are frequent outcomes of IVF.^17,18^

Because ECHO includes some autism-enriched cohorts, the prevalence of ASD in our full sample was higher than in the general population. The prevalence of ADHD was slightly lower, likely due to the higher proportion of preschool-aged children. When restricted to cohorts with data on infertility treatment type, in which the prevalence of ASD was commensurate with that in the general population, our results were consistent with our main findings of associations of subfecundity with higher externalizing problem scores and odds of ASD among children conceived naturally, although slightly attenuated and with wider CIs. Differences between our main analysis and our sensitivity analysis restricted to nonspecialized cohorts suggest that our inclusion of autism and preterm birth cohorts may have resulted in some residual genetic confounding. Differences between our main analysis and our sensitivity analysis restricted to complete cases may indicate that covariate data were not missing completely at random.

Some of our findings were not consistent with prior studies. In particular, we observed that children born to parents who conceived via non-IVF infertility treatment had higher odds of ADHD compared with children born to those who conceived naturally, regardless of whether they had a history of subfecundity. This contrasts with the results of the Groningen ART cohort study,^19^ which also compared offspring neurodevelopmental outcomes across 4 conception groups. They found no differences in neurodevelopment in infancy^19^ but detected associations of IVF with ovarian stimulation, natural IVF, and subfecundity with suboptimal neurodevelopment at ages 2 and 4 years compared with those who conceived naturally within 12 months.^20,21,22,23^ In addition to differences in demographics and IVF access between their cohort and ours, the Groningen ART study^19^ measured end points via the Hempel neurological assessment that do not align with those in our analysis. Finally, they also had a much smaller sample size (316 participants), which could account for their overall lack of statistically significant findings.

An analysis of data from 3 Danish cohorts^24^ found that children born after infertility treatment had higher total problem scores using the SDQ, but only as reported by their teachers. When Zhu et al^24^ analyzed scores reported by the mothers—as we did—or by children themselves, they observed no associations. The Danish study by Zhu et al^24^ evaluated behavior in children aged 7 to 21 years, in contrast to the younger age range in our study, which may account for their null findings. With increasing child age, any effects of subfecundity or infertility treatment may be diluted by intervening influences or life events, which were not controlled for in their study.

In the Upstate KIDS Study, which included children in New York State and assessed neurodevelopmental outcomes via the Ages and Stages Questionnaire^25^ through age 36 months, IVF use was associated with higher risk of failing in several developmental domains.^26^ While most results were no longer statistically significant when stratified by plurality, the odds of failure for gross motor and problem-solving skills were still elevated for singletons. In addition to assessing children at a younger age and for different outcomes than we did, the Upstate KIDS study^26^ did not address the issue of underlying subfecundity, as the reference group was simply participants who had not used infertility treatment, regardless of their subfecundity status. In a follow-up study assessing psychological and behavioral outcomes measured at ages 8 to 10 years, both underlying subfecundity (time to pregnancy >12 months) and infertility treatment were associated with higher risk of maternal-reported anxiety or depression in the offspring.^27^

Our observation of higher odds of ADHD among children conceived via non-IVF treatment even when compared with children conceived naturally whose parents had a history of subfecundity appears not to support the theory that underlying subfecundity explains the excess risk of adverse child neurodevelopment among children conceived via infertility treatment. However, we suspect that this finding reflects confounding by indication—specifically, PCOS. PCOS has been linked to a range of child cognitive and behavioral outcomes, but particularly ADHD. Indeed, among the 4 exposure groups included in our analysis, the highest prevalences of PCOS were in the non-IVF (49%) and IVF (31%) treatment groups, while only 11% of mothers who conceived naturally with subfecundity and 3% of mothers who conceived naturally without subfecundity had PCOS. Two other large cohort studies, the Nurses’ Health Study II^28^ and a populationwide Danish study,^29^ identified elevated odds of ASD among users of various non-IVF infertility treatments but not IVF; neither examined associations with ADHD.

### Strengths and Limitations

The primary strength of this analysis was its ability to distinguish among different infertility treatment types and tailor comparison groups that could disentangle underlying subfecundity from treatment, thereby addressing the problem of confounding by indication that plagues most studies of infertility treatment and health outcomes. In contrast with prior studies that have defined subfecundity among natural conceptions as a time to pregnancy of more than 12 months for the index pregnancy or infertility treatment for a prior pregnancy, our definition included additional indications, including repeated miscarriage, ever having tried to conceive for at least 12 months, and having had a prior consultation for or diagnosis of infertility. The size of the ECHO cohort and the richness of data available also permitted us to adjust for a host of key covariates not commonly available in registry studies.

This study also has some limitations. An important consideration when conducting infertility treatment research in the US is differential access to diagnosis and treatment, which in the case of this analysis may lead to selection bias if these factors are associated with offspring risk of neurodevelopmental problems or access to diagnosis of ASD or ADHD. Indeed, we observed demographic differences between those who conceived naturally and those who conceived via infertility treatment and adjusted for them in our analysis, but the possibility of residual confounding remains. In addition, not all exposure variables were assessed across all cohorts, such that the analytic samples for our 2 sets of regression analyses were not only different sizes but included different mixes of participants, although their demographic and health characteristics were comparable. While the overrepresentation of children with ASD and, to a lesser degree, preterm births in our sample may limit the generalizability of our results, we are particularly reassured by the consistency with our main results of the sensitivity analysis that excluded the largest autism-enriched cohort and had an ASD prevalence closer to that in the general population.

## Conclusions

In this ECHO Cohort study, subfecundity was associated with offspring behavior problems and ASD regardless of infertility treatment, and non-IVF infertility treatment was associated with child ADHD. Future studies are needed to identify specific causes of subfecundity and indications for infertility treatment that may explain these results and help to elucidate biological mechanisms.

## Notes

Supplement 1. eFigure. Directed acyclic graph of hypothesized relations among exposures, outcomes, and covariates. eTable 1. Associations of subfecundity with neurodevelopmental outcomes, with imputation; General population cohorts only. eTable 2. Associations of subfecundity with neurodevelopmental outcomes: all pregnancies with available information on fertility treatment, with imputation; General population cohorts only eTable 3. Associations of subfecundity with neurodevelopmental outcomes, complete case analysis. eTable 4. Associations of non-IVF treatment and IVF treatment with neurodevelopmental outcomes: all pregnancies with available information on fertility treatment, complete case analysis. eTable 5. Associations of subfecundity with neurodevelopmental outcomes among those who conceived naturally: full sample and sample restricted to cohorts with information on fertility treatment type. eTable 6. Indirect effect estimates of statistically significant associations between subfecundity and child neurodevelopment mediated by gestational age at birth.

Supplement 2. Members of the ECHO Cohort Consortium

Supplement 3. Data Sharing Statement
