# Supplementary material for: Subfecundity, Infertility Treatment, and Child Neurodevelopment
Source: JAMA Netw Open. 2026 Jun 8;9(6):e2617324. doi: 10.1001/jamanetworkopen.2026.17324 (PMC13247815; doi:10.1001/jamanetworkopen.2026.17324)
Supplement: Supplement 1. — eFigure. Directed acyclic graph of hypothesized relations among exposures, outcomes, and covariates. eTable 1. Associations of subfecundity with neurodevelopmental outcomes, with imputation; General population cohorts only. eTable 2. Associations of subfecundity with neurodevelopmental outcomes: all pregnancies with available information on fertility treatment, with imputation; General population cohorts only eTable 3. Associations of subfecundity with neurodevelopmental outcomes, complete case analysis. eTable 4. Associations of non-IVF treatment and IVF treatment with neurodevelopmental outcomes: all pregnancies with available information on fertility treatment, complete case analysis. eTable 5. Associations of subfecundity with neurodevelopmental outcomes among those who conceived naturally: full sample and sample restricted to cohorts with information on fertility treatment type. eTable 6. Indirect effect estimates of statistically significant associations between subfecundity and child neurodevelopment mediated by gestational age at birth. [file jamanetwopen-e2617324-s001.pdf]

Supplemental Online Content

Kahn LG, Hipwell AE, Stanford JB, et al; the ECHO Cohort Consortium. Subfecundity, infertility treatment, and child neurodevelopment. *JAMA Netw Open*. 2026;9(6):e2617324. doi:10.1001/jamanetworkopen.2026.17324

eFigure. Directed acyclic graph of hypothesized relations among exposures, outcomes, and covariates

eTable 1. Associations of subfecundity with neurodevelopmental outcomes, with imputation; General population cohorts only

eTable 2. Associations of subfecundity with neurodevelopmental outcomes: all pregnancies with available information on fertility treatment, with imputation

eTable 3. Associations of subfecundity with neurodevelopmental outcomes: complete case analysis

eTable 4. Associations of non-IVF treatment and IVF treatment with neurodevelopmental outcomes: all pregnancies with available information on fertility treatment: complete case analysis

eTable 5. Associations of subfecundity with neurodevelopmental outcomes among those who conceived naturally: full sample and sample restricted to cohorts with information on fertility treatment type

eTable 6. Indirect effect estimates of statistically significant associations between subfecundity and child neurodevelopment mediated by gestational age at birth

This supplemental material has been provided by the authors to give readers additional information about their work.

© 2026 Kahn LG et al. *JAMA Network Open*.

eFigure. Directed acyclic graph of hypothesized relations among exposures, outcomes, and covariates.

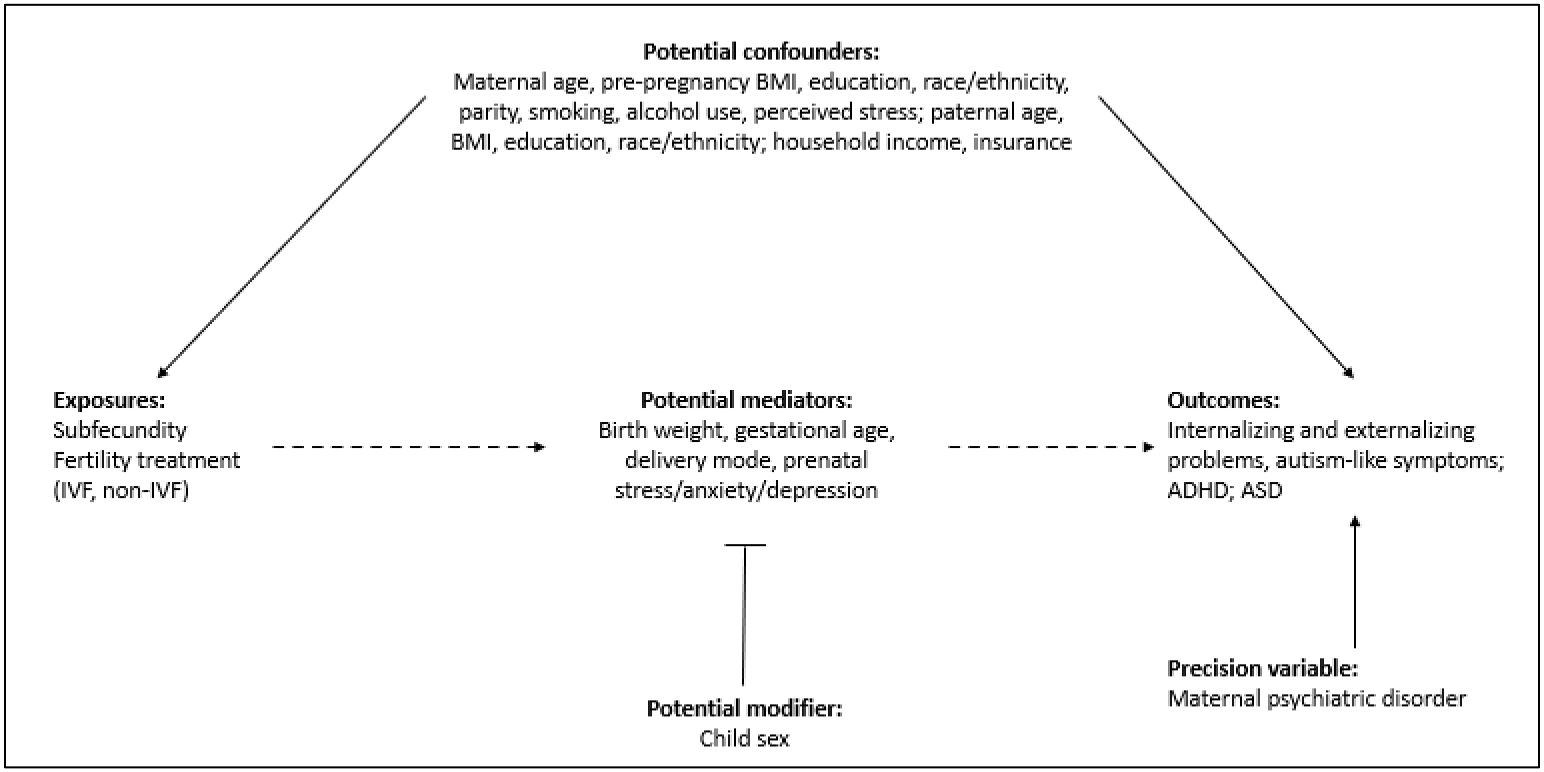

© 2026 Kahn LG et al. *JAMA Network Open*.

eTable 1. Associations of subfecundity with neurodevelopmental outcomes, with imputation; General population cohorts only.

	**All Pregnancies Subfecundity vs. no subfecundity**	**Among natural conceptions, subfecundity vs. no subfecundity**
Continuous outcomes (Linear models)[a]	beta (95% CI)	beta (95% CI)
	(n=9522, 1776 vs. 7746)	(n=8983, 1238 vs. 7745)
Externalizing Behavior Score[b]	0.31 (-0.04, 0.66)	0.16 (-0.24, 0.56)
Internalizing Behavior Score[b]	0.29 (-0.06, 0.64)	0.20 (-0.20, 0.60)
	(n=8525, 2686 vs. 6839)	(n=8001, 1173 vs. 6828)
Social Responsiveness Scale Score[b]	0.90 (-0.17, 1.97)	0.63 (-0.61, 1.87)
Categorical outcomes (Logistic models)[a]	aOR (95% CI)	aOR (95% CI)
	(n=9442, 1859 vs. 7583)	(n=8886, 1304 vs. 7582)
Attention-Deficit/Hyperactivity Disorder Diagnosis	0.99 (0.82, 1.18)	0.84 (0.67, 1.05)
Autism Spectrum Disorder Diagnosis	1.10 (0.87, 1.41)	1.13 (0.77, 1.65)

[a] All models are adjusted for child sex; maternal age, race, ethnicity, education, parity, pre-pregnancy BMI, tobacco use during index pregnancy, alcohol use during index pregnancy, and lifetime psychiatric diagnosis; and household income; with additional adjustment of child age for continuous outcomes.

[b] Externalizing and internalizing problem scores are based on the harmonization of Child Behavior Checklist and Strengths and Difficulties Questionnaire data; the Social Responsiveness Scale score is the total raw score.

© 2026 Kahn LG et al. *JAMA Network Open*.

eTable 2. Associations of subfecundity with neurodevelopmental outcomes: all pregnancies with available information on fertility treatment, with imputation; General population cohorts only.

For each linear model, the table entries give the sample size of the comparison groups and the average estimated difference in scores (Beta) between the subfecundity and no subfecundity groups with 95% confidence intervals. Logistic model results are shown as odds ratios (OR) with 95% confidence intervals (CI).

**Outcomes**	**Vs. Natural conception without subfecundity**		**Vs. Natural conception with subfecundity**	
	**Non-IVF treatment**	**IVF treatment**	**Non-IVF treatment**	**IVF treatment**
**Continuous outcomes (Linear models)[a]**	**b (95% CI), n**	**b (95% CI), n**	**b (95% CI), n**	**b (95% CI), n**
	(n=6504, 299 vs. 6205)	(n=6435, 230 vs. 6205)	(n=1475, 299 vs. 1176)	(n=1406, 230 vs. 1176)
Externalizing Behavior Score[b]	0.48 (-0.26, 1.22)	0.77 (-0.08, 1.63)	0.40 (-0.41, 1.20)	0.69 (-0.23, 1.60)
Internalizing Behavior Score[b]	0.36 (-0.39, 1.10)	0.65 (-0.20, 1.50)	0.19 (-0.61, 0.99)	0.48 (-0.43, 1.40)
Social Responsiveness Scale Score[b]	1.78 (-0.45, 4.02)	0.38 (-2.27, 3.04)	1.28 (-1.14, 3.69)	-0.12 (-2.95, 2.71)
**Categorical outcomes (Logistic models)[a]**	**aOR (95% CI), n**	**aOR (95% CI), n**	**aOR (95% CI), n**	**aOR (95% CI), n**
	(n=6555, 342 vs. 6213)	(n=6418, 205 vs. 6213)	(n=1611, 342 vs. 1269)	(n=1474, 205 vs. 1269)
Attention-Deficit/Hyperactivity Disorder Diagnosis	1.46 (0.94, 2.27)	1.19 (0.57, 2.45)	**1.65 (1.01, 2.71)**	1.34 (0.62, 2.88)
Autism Spectrum Disorder Diagnosis	0.97 (0.46, 2.03)	0.93 (0.33, 2.59)	0.86 (0.39, 1.91)	0.83 (0.28, 2.40)

[a] All models are adjusted for child sex, maternal age, race, ethnicity, education, parity, pre-pregnancy BMI, tobacco use during index pregnancy, alcohol use during index pregnancy, and lifetime psychiatric diagnosis; and household income; with additional adjustment of child age for continuous outcomes.

[b] Externalizing and internalizing problem scores are based on the harmonization of Child Behavior Checklist and Strengths and Difficulties Questionnaire data; the Social Responsiveness Scale score is the total raw score.

© 2026 Kahn LG et al. *JAMA Network Open*.

eTable 3. Associations of subfecundity with neurodevelopmental outcomes, complete case analysis.

	**All Pregnancies Subfecundity vs. no subfecundity**	**Among natural conceptions, subfecundity vs. no subfecundity**
Continuous outcomes (Linear models)[a]	beta (95% CI)	beta (95% CI)
	(n=6461, 1506 vs. 4955)	(n=6039, 1088 vs. 4951)
Externalizing Behavior Score[b]	**0.56 (0.16, 0.97)**	**0.54 (0.09, 1.00)**
Internalizing Behavior Score[b]	0.36 (-0.05, 0.77)	0.21 (-0.25, 0.67)
	(n=6126, 1455 vs. 4671)	(n=5711, 1044 vs. 4667)
Social Responsiveness Scale Score[b]	0.36 (-0.90, 1.62)	-0.05 (-1.47, 1.37)
Categorical outcomes (Logistic models)[a]	aOR (95% CI)	aOR (95% CI)
	(n=7011, 1667 vs. 5344)	(n=6542, 1201 vs. 5341)
Attention-Deficit/Hyperactivity Disorder Diagnosis	1.06 (0.83, 1.37)	0.90 (0.68, 1.21)
Autism Spectrum Disorder Diagnosis	1.14 (0.82, 1.57)	1.11 (0.78, 1.58)

[a] All models are adjusted for child sex; maternal age, race, ethnicity, education, parity, pre-pregnancy BMI, tobacco use during index pregnancy, alcohol use during index pregnancy, and lifetime psychiatric diagnosis; and household income; with additional adjustment of child age for continuous outcomes.

[b] Externalizing and internalizing problem scores are based on the harmonization of Child Behavior Checklist and Strengths and Difficulties Questionnaire data; the Social Responsiveness Scale score is the total raw score.

© 2026 Kahn LG et al. *JAMA Network Open*.

eTable 4. Associations of non-IVF treatment and IVF treatment with neurodevelopmental outcomes: all pregnancies with available information on fertility treatment, complete case analysis.

For each linear model, the table entries give the sample size of the comparison groups and the average estimated difference in scores (Beta) between the subfecundity and no subfecundity groups with 95% confidence intervals. Logistic model results are shown as odds ratios (OR) with 95% confidence intervals (CI).

Outcomes	Vs. Natural conception without subfecundity		Vs. Natural conception with subfecundity	
	Non-IVF treatment	IVF treatment	Non-IVF treatment	IVF treatment
**Continuous outcomes (Linear models)[a]**	**b (95% CI), n**	**b (95% CI), n**	**b (95% CI), n**	**b (95% CI), n**
	(n=4095, 222 vs. 3873)	(n=4055, 182 vs. 3873)	(n=1224, 222 vs. 1002)	(n=1184, 182 vs. 1002)
Externalizing Behavior Score[b]	0.86 (-0.04, 1.76)	0.28 (-0.73, 1.29)	0.14 (-0.82, 1.10)	-0.44 (-1.50, 0.63)
Internalizing Behavior Score[b]	0.86 (-0.05, 1.76)	0.71 (-0.30, 1.72)	0.52 (-0.44, 1.48)	0.37 (-0.70, 1.43)
	(n=4032, 232 vs. 3800)	(n=3966, 166 vs. 3800)	(n=1209, 232 vs. 977)	(n=1143, 166 vs. 977)
Social Responsiveness Scale Score[b]	2.42 (-0.19, 5.02)	0.38 (-2.73, 3.48)	2.20 (-0.58, 4.97)	0.16 (-3.11, 3.42)
**Categorical outcomes (Logistic models)[a]**	**aOR (95% CI), n**	**aOR (95% CI), n**	**aOR (95% CI), n**	**aOR (95% CI), n**
	(n=4597, 267 vs. 4330)	(n=4514, 184 vs. 4330)	(n=1384 267 vs. 1117)	(n=1301, 184 vs. 1117)
Attention-Deficit/Hyperactivity Disorder Diagnosis	**1.75 (1.09, 2.80)**	1.56 (0.80, 3.03)	**2.02 (1.21, 3.37)**	1.80 (0.90, 3.60)
Autism Spectrum Disorder Diagnosis	1.44 (0.66, 3.15)	1.14 (0.39, 3.34)	1.36 (0.59, 3.13)	1.07 (0.35, 3.29)

[a] All models are adjusted for child sex, maternal age, race, ethnicity, education, parity, pre-pregnancy BMI, tobacco use during index pregnancy, alcohol use during index pregnancy, and lifetime psychiatric diagnosis; and household income; with additional adjustment of child age for continuous outcomes.

[b] Externalizing and internalizing problem scores are based on the harmonization of Child Behavior Checklist and Strengths and Difficulties Questionnaire data; the Social Responsiveness Scale score is the total raw score.

© 2026 Kahn LG et al. *JAMA Network Open*.

eTable 5. Associations of subfecundity with neurodevelopmental outcomes among those who conceived naturally: full sample and sample restricted to cohorts with information on fertility treatment type.

For each linear model, the table entries give the sample size of the comparison groups and the average estimated difference in scores (Beta) between the subfecundity and no subfecundity groups with 95% confidence intervals. Logistic model results are shown as odds ratios (OR) with 95% confidence intervals (CI).

**Outcome**	**Natural Conceptions Pregnancies, Subfecundity vs. no subfecundity (full sample)**		**Natural Conceptions Pregnancies, Subfecundity vs. no subfecundity (restricted sample)**	
**Continuous outcomes** (Linear models)[a]	N in the model	Adj Beta (95% CI)	N in the model	Adj Beta (95% CI)
Externalizing Problem Score[b]	n=10358, 1640 vs. 8718	**0.45 (0.07, 0.83)**	n=8229, 1435 vs. 6794	0.40 (-0.00, 0.80)
Internalizing Problem Score[b]		0.14 (-0.23, 0.52)		0.13 (-0.26, 0.54)
Social Responsiveness Scale Score[b]	n=9118, 1507 vs. 7611	1.12 (-0.09, 2.34)	n=7346, 1361 vs. 5985	0.79 (-0.43, 2.01)
**Categorical outcomes** (Logistic models)[a]	N in the model	aOR (95% CI)	N in the model	aOR (95% CI)
Attention-Deficit/Hyperactivity Disorder Diagnosis	n=10807, 1785 vs. 9022	0.91 (0.73, 1.12)	n=8370, 1525 vs. 6845	0.87 (0.67, 1.13)
Autism Spectrum Disorder Diagnosis	n=10807, 1785 vs. 9022	**1.31 (1.04, 1.64)**	n=8370, 1525 vs. 6845	1.20 (0.88, 1.65)

[a] All models are adjusted for child sex, maternal age, race, ethnicity, education, parity, pre-pregnancy BMI, tobacco use during index pregnancy, alcohol use during index pregnancy, and lifetime psychiatric diagnosis; and household income; with additional adjustment of child age for continuous outcomes.

[b] Externalizing and internalizing problem scores are based on the harmonization of Child Behavior Checklist and Strengths and Difficulties Questionnaire data; the Social Responsiveness Scale score is the total raw score.

© 2026 Kahn LG et al. *JAMA Network Open*.

eTable 6. Indirect effect estimates of statistically significant associations between subfecundity and child neurodevelopment mediated by gestational age at birth.

Outcome	All Pregnancies<br>Subfecundity vs. no subfecundity		Natural Conceptions Pregnancies,<br>Subfecundity vs. no subfecundity	
**Continuous outcomes** (Linear models)[a]	N in the model	Adj Beta (95% CI)	N in the model	Adj Beta (95% CI)
Externalizing Problem Score[b]	n=10997,<br>2274 vs. 8723	**0.03 (0.01, 0.05)**	n=10358,<br>1640 vs. 8718	**0.02 (0.00, 0.04)**
Social Responsiveness Scale Score[b]	n=9717,<br>2101 vs. 7616	**0.07 (0.01, 0.12)**		
**Categorical outcomes** (Logistic models)[a]	N in the model	aOR (95% CI)	N in the model	aOR (95% CI)
Autism Spectrum Disorder Diagnosis	n=11455,<br>2429 vs. 9026	1.00 (1.00, 1.00)	n=10807,<br>1785 vs. 9022	1.00 (1.00, 1.00)

[a] All models are adjusted for child sex; maternal age, race, ethnicity, education, parity, pre-pregnancy BMI, tobacco use during index pregnancy, alcohol use during index pregnancy, and lifetime psychiatric diagnosis; and household income; with additional adjustment of child age for continuous outcomes.

[b] Externalizing problem scores are based on the harmonization of Child Behavior Checklist and Strengths and Difficulties Questionnaire data; the Social Responsiveness Scale score is the total raw score.

© 2026 Kahn LG et al. *JAMA Network Open*.
