# Supplementary material for: Subfecundity, Infertility Treatment, and Child Neurodevelopment
Source: JAMA Netw Open. 2026 Jun 8;9(6):e2617324. doi: 10.1001/jamanetworkopen.2026.17324 (PMC13247815; doi:10.1001/jamanetworkopen.2026.17324)
Supplement: Supplement 2. — Members of the ECHO Cohort Consortium [file jamanetwopen-e2617324-s002.pdf]

*First name, last name, and suffix (if applicable) are required and will appear in PubMed.

*Group Name(s): ECHO Collaborators							
***First Name and Middle Initial(s)**	***Last Name**	***Suffix (eg, Jr, III)**	Academic Degrees	Institution	Location (city, state/province, country)	Role or Contribution, eg, chair, principal investigator	Group (if more than 1 Group listed in the byline) and/or Subgroup (eg, Steering Committee)
L Kristin	Newby		MD, MHS	Division of Cardiology, Department of Medicine	Duke Clinical Research Institute, Duke University School of Medicine	Durham, North Carolina, USA	ECHO Coordinating Center Principal Investigator
Linda	Adair		PhD	Department of Nutrition	Gillings School of Global Public Health, University of North Carolina at Chapel Hill	Chapel Hill, North Carolina, USA	ECHO Coordinating Center Principal Investigator
Lisa P.	Jacobson		ScD	Department of Epidemiology	Johns Hopkins University, Bloomberg School of Public Health	Baltimore, Maryland, USA	ECHO Data Analysis Center Principal Investigator
Diane	Catellier		DrPH	N/A	Research Triangle Institute	Research Triangle Park, North Carolina, USA	ECHO Data Analysis Center Principal Investigator
Monica	McGrath		ScD	Department of Epidemiology	Johns Hopkins University, Bloomberg School of Public Health	Baltimore, Maryland, USA	ECHO Johns Hopkins University Data Analysis Center Director Co-Investigator
Christian	Douglas		DrPH	N/A	Research Triangle Institute	Research Triangle Park, North Carolina, USA	ECHO RTI Data Analysis Center Director Co-Investigator
Priya	Duggal		PhD	Department of Epidemiology	Johns Hopkins University, Bloomberg School of Public Health	Baltimore, Maryland, USA	ECHO Data Analysis Center Genetics Methods Lead, Co-Investigator
Emily	Knapp		PhD	Department of Epidemiology	Johns Hopkins University, Bloomberg School of Public Health	Baltimore, Maryland, USA	ECHO Data Analysis Center Co-Investigator

Supplemental Online Content: Nonauthor Collaborators
*First name, last name, and suffix (if applicable) are required and will appear in PubMed.

***First Name and Middle Initial(s)**	***Last Name**	***Suffix (eg, Jr, III)**	Academic Degrees	Institution	Location (city, state/province, country)	Role or Contribution, eg, chair, principal investigator	Group (if more than 1 Group listed in the byline) and/or Subgroup (eg, Steering Committee)
Amii M.	Kress		PhD	Department of Epidemiology	Johns Hopkins University, Bloomberg School of Public Health	Baltimore, Maryland, USA	ECHO Data Analysis Center General Methods Co-Investigator
Courtney K.	Blackwell		PhD	Department of Medical Social Sciences	Feinberg School of Medicine, Northwestern University	Chicago, Illinois, USA	Measurement Core Co-Investigator
Maxwell A.	Mansolf		PhD	Department of Medical Social Sciences	Feinberg School of Medicine, Northwestern University	Chicago, Illinois, USA	Measurement Core Co-Investigator
Jin-Shei	Lai		PhD	Department of Medical Social Sciences	Feinberg School of Medicine, Northwestern University	Chicago, Illinois, USA	Measurement Core Co-Investigator
Emily	Ho		PhD	Department of Medical Social Sciences	Feinberg School of Medicine, Northwestern University	Chicago, Illinois, USA	Measurement Core Co-Investigator
David	Cella		PhD	Department of Medical Social Sciences	Feinberg School of Medicine, Northwestern University	Chicago, Illinois, USA	Measurement Core Principal Investigator
Richard	Gershon		PhD	Department of Medical Social Sciences	Feinberg School of Medicine, Northwestern University	Chicago, Illinois, USA	Measurement Core Principal Investigator
Michelle L.	Macy		MD	Department of Pediatrics	Feinberg School of Medicine, Northwestern University and Ann & Robert H. Lurie Children's Hospital of Chicago	Chicago, Illinois, USA	Measurement Core Co-Investigator
Suman R.	Das		PhD	Division of Infectious Diseases, Department of Medicine	Vanderbilt University Medical Center	Nashville, Tennessee, USA	ECHO Laboratory Core Principal Investigator

Supplemental Online Content: Nonauthor Collaborators
*First name, last name, and suffix (if applicable) are required and will appear in PubMed.

***First Name and Middle Initial(s)**	***Last Name**	***Suffix (eg, Jr, III)**	Academic Degrees	Institution	Location (city, state/province, country)	Role or Contribution, eg, chair, principal investigator	Group (if more than 1 Group listed in the byline) and/or Subgroup (eg, Steering Committee)
Jane E.	Freedman		MD	Division of Cardiovascular Medicine, Department of Medicine	Vanderbilt University Medical Center	Nashville, Tennessee, USA	ECHO Laboratory Core Principal Investigator
Simon A.	Mallal		MBBS	Division of Infectious Diseases, Department of Medicine	Vanderbilt University Medical Center	Nashville, Tennessee, USA	ECHO Laboratory Core Principal Investigator
John A.	McLean		PhD	Department of Chemistry	Vanderbilt University	Nashville, Tennessee, USA	ECHO Laboratory Core Principal Investigator
Ravi V.	Shah		MD	Division of Cardiovascular Medicine, Department of Medicine	Vanderbilt University Medical Center	Nashville, Tennessee, USA	ECHO Laboratory Core Principal Investigator
Meghan H.	Shilts		MHS	Division of Infectious Diseases, Department of Medicine	Vanderbilt University Medical Center	Nashville, Tennessee, USA	ECHO Laboratory Core Principal Investigator Admin Designee
Akram N.	Alshawabkeh		PhD	College of Engineering	Northeastern University	Boston, Massachusetts, USA	ECHO Cohort Study Site Principal Investigator
Jose F.	Cordero		MD	College of Public Health, Department of Epidemiology & Biostatistics	University of Georgia	Athens, Georgia; USA	ECHO Cohort Study Site Co-Director
John	Meeker		ScD	Environmental Health Sciences, School of Public Health	University of Michigan	Ann Arbor, Michigan; USA	ECHO Cohort Study Site Co-Director
Leonardo	Trasande		MD, MPP	Departments of Pediatrics and Population Health	NYU Grossman School of Medicine	New York, New York, USA	ECHO Cohort Study Site Principal Investigator
Carlos A.	Camargo	Jr.	MD, DrPH	Department of Emergency Medicine	Massachusetts General Hospital, Harvard Medical School	Boston, Massachusetts, USA	ECHO Cohort Study Site Principal Investigator
Kohei	Hasegawa		MD, PhD	Department of Emergency Medicine	Massachusetts General Hospital, Harvard Medical School	Boston, Massachusetts, USA	ECHO Cohort Study Site Co-Investigator
Zhaozhong	Zhu		ScD	Department of Emergency Medicine	Massachusetts General Hospital, Harvard Medical School	Boston, Massachusetts, USA	ECHO Cohort Study Site Co-Investigator

*First name, last name, and suffix (if applicable) are required and will appear in PubMed.

***First Name and Middle Initial(s)**	***Last Name**	***Suffix (eg, Jr, III)**	Academic Degrees	Institution	Location (city, state/province, country)	Role or Contribution, eg, chair, principal investigator	Group (if more than 1 Group listed in the byline) and/or Subgroup (eg, Steering Committee)
Ashley F.	Sullivan		MS, MPH	Department of Emergency Medicine	Massachusetts General Hospital, Harvard Medical School	Boston, Massachusetts, USA	ECHO Cohort Study Site Award Project Director
Dana	Dabelea		MD, PhD	Lifecourse Epidemiology of Adiposity and Diabetes (LEAD) Center	University of Colorado Anschutz Medical Campus	Aurora, Colorado, USA	ECHO Cohort Study Site Principal Investigator
Wei	Perng		PhD, MPH	Lifecourse Epidemiology of Adiposity and Diabetes (LEAD) Center	University of Colorado Anschutz Medical Campus	Aurora, Colorado, USA	ECHO Cohort Study Site Principal Investigator
Traci A.	Bekelman		PhD, MPH	Lifecourse Epidemiology of Adiposity and Diabetes (LEAD) Center	University of Colorado Anschutz Medical Campus	Aurora, Colorado, USA	ECHO Cohort Study Site Principal Investigator
Greta	Wilkening		PhD, MPH	Lifecourse Epidemiology of Adiposity and Diabetes (LEAD) Center	University of Colorado Anschutz Medical Campus	Aurora, Colorado, USA	ECHO Cohort Study Site Co-Investigator
Sheryl	Magzamen		PhD	Environmental and Radiological Health Sciences	Colorado School of Public Health, Colorado State University	Fort Collins, Colorado, USA	ECHO Cohort Study Site Co-Investigator
Brianna F.	Moore		PhD, MS	Lifecourse Epidemiology of Adiposity and Diabetes (LEAD) Center	University of Colorado Anschutz Medical Campus	Aurora, Colorado, USA	ECHO Cohort Study Site Principal Investigator
Anne P.	Starling		PhD	Epidemiology	University of North Carolina at Chapel Hill	Chapel Hill, North Carolina, USA	ECHO Cohort Study Site Principal Investigator
Deborah J.	Rinehart		PhD	Center for Health Systems Research	Denver Health and Hospital Authority	Denver, Colorado, USA	ECHO Cohort Study Site Co-Investigator
Daphne	Koinis Mitchell		Ph.D	Department of Pediatrics	Rhode Island Hospital, The Alpert Medical School of Brown University	Providence, Rhode Island, USA	ECHO Cohort Study Site Principal Investigator

*First name, last name, and suffix (if applicable) are required and will appear in PubMed.

*First Name and Middle Initial(s)	*Last Name	*Suffix (eg, Jr, III)	Academic Degrees	Institution	Location (city, state/province, country)	Role or Contribution, eg, chair, principal investigator	Group (if more than 1 Group listed in the byline) and/or Subgroup (eg, Steering Committee)
Viren	D'Sa		MD	Department of Pediatrics	Rhode Island Hospital, The Alpert Medical School of Brown University	Providence, Rhode Island, USA	ECHO Cohort Study Site Principal Investigator
Sean C.L.	Deoni		PhD	Division of Gender Equality, Maternal, Newborn & Child Health Discovery & Tools Team	Bill & Melinda Gates Foundation	Seattle, Washington, USA	ECHO Cohort Study Site Principal Investigator
Hans-Georg	Mueller		PhD	Department of Statistics	University of California, Davis	Davis, California, USA	ECHO Cohort Study Site Co-Investigator
Cristiane S.	Duarte		PhD, MPH	Division of Child and Adolescent Psychiatry	Columbia University - NYSPI	New York, New York, USA	ECHO Cohort Study Site Principal Investigator
Catherine	Monk		PhD	Department of Obstetrics & Gynecology	Columbia University - NYSPI	New York, New York, USA	ECHO Cohort Study Site Principal Investigator
Glorisa	Canino		PhD	Behavioral Sciences Research Institute	University of Puerto Rico, School of Medicine	Rio Piedras, Puerto Rico	ECHO Cohort Study Site Principal Investigator
Jonathan	Posner		MD	Child & Family Mental Health & Community Psychiatry Division	Duke University School of Medicine, Duke Psychiatry & Behavioral Sciences	Durham, North Carolina, USA	ECHO Cohort Study Site Principal Investigator
Tenneill	Murray		MPH	Division of Child and Adolescent Psychiatry	Columbia University - NYSPI	New York, New York, USA	ECHO Cohort Study Site Co-Director
Claudia	Lugo-Candelas		PhD	Division of Child and Adolescent Psychiatry	Columbia University - NYSPI	New York, New York, USA	ECHO Cohort Study Site Principal Investigator
Anne L.	Dunlop		MD, MPH	Department of Gynecology and Obstetrics	Emory University School of Medicine	Atlanta, Georgia, USA	ECHO Cohort Study Site Principal Investigator
Patricia A.	Brennan		PhD	Department of Psychology	Emory University	Atlanta, Georgia, USA	ECHO Cohort Study Site Principal Investigator
Christine	Hockett		PhD	N/A; Department of Pediatrics	Avera Research Institute; University of South Dakota School of Medicine	Rapid City, South Dakota, USA; Sioux Falls, South Dakota, USA	ECHO Cohort Study Site Principal Investigator

Supplemental Online Content: Nonauthor Collaborators
*First name, last name, and suffix (if applicable) are required and will appear in PubMed.

***First Name and Middle Initial(s)**	***Last Name**	***Suffix (eg, Jr, III)**	Academic Degrees	Institution	Location (city, state/province, country)	Role or Contribution, eg, chair, principal investigator	Group (if more than 1 Group listed in the byline) and/or Subgroup (eg, Steering Committee)
Amy	Elliott		PhD	N/A; Department of Pediatrics	Avera Research Institute ; University of South Dakota School of Medicine	Sioux Falls, South Dakota, USA	ECHO Cohort Study Site Principal Investigator
Assiamira	Ferrara		MD, PhD	Division of Research	Kaiser Permanente Northern California	Oakland, California, USA	ECHO Cohort Study Site Principal Investigator
Lisa A.	Croen		PhD	Division of Research	Kaiser Permanente Northern California	Oakland, California, USA	ECHO Cohort Study Site Principal Investigator
Monique M.	Hedderson		PhD	Division of Research	Kaiser Permanente Northern California	Oakland, California, USA	ECHO Cohort Study Site Principal Investigator
John	Ainsworth		PhD	Centre for Health Informatics	University of Manchester	Manchester, United Kingdom	ECHO Cohort Study Site Principal Investigator
Leonard B.	Bacharier		MD	Department of Pediatrics, Monroe Carell Jr Children's Hospital at Vanderbilt	Vanderbilt University Medical Center	Nashville, Tennessee, USA	ECHO Cohort Study Site Principal Investigator
Casper G.	Bendixsen		PhD	National Farm Medicine Center	Marshfield Clinic Research Institute	Marshfield, Wisconsin, USA	ECHO Cohort Study Site Principal Investigator
James E.	Gern		MD	Department of Pediatrics	University of Wisconsin School of Medicine and Public Health	Madison, Wisconsin, USA	ECHO Cohort Study Site Principal Investigator
Diane R.	Gold		MD	The Channing Division of Network Medicine; Department of Medicine	Brigham and Women's Hospital; Harvard Medical School	Boston, Massachusetts, USA	ECHO Cohort Study Site Principal Investigator
Tina V.	Hartert		MD, MPH	Division of Pediatric Allergy, Immunology, and Pulmonary Medicine, Department of Medicine, Department of Pediatrics	Vanderbilt University Medical Center	Nashville, Tennessee, USA	ECHO Cohort Study Site Principal Investigator
Daniel J.	Jackson		MD	Department of Pediatrics	University of Wisconsin School of Medicine and Public Health	Madison, Wisconsin, USA	ECHO Cohort Study Site Principal Investigator

Supplemental Online Content: Nonauthor Collaborators
*First name, last name, and suffix (if applicable) are required and will appear in PubMed.

***First Name and Middle Initial(s)**	***Last Name**	***Suffix (eg, Jr, III)**	Academic Degrees	Institution	Location (city, state/province, country)	Role or Contribution, eg, chair, principal investigator	Group (if more than 1 Group listed in the byline) and/or Subgroup (eg, Steering Committee)
Christine C.	Johnson		PhD	Department of Public Health Sciences	Henry Ford Health	Detroit, Michigan, USA	ECHO Cohort Study Site Principal Investigator
Christine L.M.	Joseph		PhD	Department of Public Health Sciences	Henry Ford Health	Detroit, Michigan, USA	ECHO Cohort Study Site Principal Investigator
Meyer	Kattan		MD	Department of Pediatrics	Columbia University Medical Center	New York, New York, USA	ECHO Cohort Study Site Principal Investigator
Gurjit K.	Khurana Hershey		MD, PhD	Division of Asthma Research	Cincinnati Children's Hospital Medical Center	Cincinnati, Ohio, USA	ECHO Cohort Study Site Principal Investigator
Robert F.	Lemanske, Jr.		MD	Department of Pediatrics	University of Wisconsin School of Medicine and Public Health	Madison, Wisconsin, USA	ECHO Cohort Study Site Principal Investigator
Susan V.	Lynch		PhD	Department of Medicine	University of California	San Francisco, California, USA	ECHO Cohort Study Site Principal Investigator
Rachel L.	Miller		MD	Department of Medicine; Division of Clinical Immunology	Icahn School of Medicine at Mount Sinai	New York, New York, USA	ECHO Cohort Study Site Principal Investigator
George T.	O'Connor		MD	Department of Pediatrics	Boston University School of Medicine	Boston, Massachusetts, USA	ECHO Cohort Study Site Principal Investigator
Carole	Ober		PhD	Department of Human Genetics	University of Chicago	Chicago, Illinois, USA	ECHO Cohort Study Site Principal Investigator
Dennis	Ownby		MD	Department of Public Health Sciences	Henry Ford Health	Detroit, Michigan, USA	ECHO Cohort Study Site Principal Investigator
Katherine	Rivera-Spoljaric		MD	Department of Pediatrics	Washington University School of Medicine	St Louis, Missouri, USA	ECHO Cohort Study Site Principal Investigator
Patrick H.	Ryan		PhD	Department of Pediatrics and College of Medicine; Division of Biostatistics and Epidemiology	University of Cincinnati	Cincinnati, Ohio, USA	ECHO Cohort Study Site Principal Investigator
Christine M.	Seroogy		MD	Department of Pediatrics	University of Wisconsin School of Medicine and Public Health	Madison, Wisconsin, USA	ECHO Cohort Study Site Principal Investigator

Supplemental Online Content: Nonauthor Collaborators
*First name, last name, and suffix (if applicable) are required and will appear in PubMed.

***First Name and Middle Initial(s)**	***Last Name**	***Suffix (eg, Jr, III)**	Academic Degrees	Institution	Location (city, state/province, country)	Role or Contribution, eg, chair, principal investigator	Group (if more than 1 Group listed in the byline) and/or Subgroup (eg, Steering Committee)
Anne Marie	Singh		MD	Department of Pediatrics	University of Wisconsin School of Medicine and Public Health	Madison, Wisconsin, USA	ECHO Cohort Study Site Principal Investigator
Robert A.	Wood		MD	Department of Pediatrics	Johns Hopkins University School of Medicine	Baltimore, Maryland, USA	ECHO Cohort Study Site Principal Investigator
Edward M.	Zoratti		MD	Division of Allergy and Clinical Immunology	Henry Ford Health	Detroit, Michigan, USA	ECHO Cohort Study Site Principal Investigator
Rima	Habre		ScD, MSc	Department of Population and Public Health Sciences	University of Southern California	Los Angeles, California, USA	ECHO Cohort Study Site Principal Investigator
Shohreh	Farzan		PhD	Department of Population and Public Health Sciences	University of Southern California	Los Angeles, California, USA	ECHO Cohort Study Site Principal Investigator
Frank D.	Gilliland		MD, MPH, PhD	Department of Population and Public Health Sciences	University of Southern California	Los Angeles, California, USA	ECHO Cohort Study Site Principal Investigator
Irva	Hertz-Picciotto		PhD	MIND Institute and Department of Public Health Sciences	University of California, Davis	Davis, California, USA	ECHO Cohort Study Site Principal Investigator
Deborah H.	Bennett		Ph.D	Department of Public Health Sciences	University of California, Davis	Davis, California, USA	ECHO Cohort Study Site Principal Investigator
Julie B.	Schweitzer		Ph.D	Department of Psychiatry and Behavioral Science and the MIND Institute	University of California, Davis	Davis, California, USA	ECHO Cohort Study Site Principal Investigator
Rebecca J.	Schmidt		Ph.D	MIND Institute and Department of Public Health Sciences	University of California, Davis	Davis, California, USA	ECHO Cohort Study Site Principal Investigator
Janine M.	LaSalle		PhD	Medical Microbiology and Immunology; MIND Institute	University of California, Davis	Davis, California, USA	ECHO Cohort Study Site Co-Investigator
Alison E.	Hipwell		PhD, ClinPsyD	Psychiatry and Psychology	University of Pittsburgh	Pittsburgh, Pennsylvania, USA	ECHO Cohort Study Site Principal Investigator
Catherine J.	Karr		MD, MS, PhD	Department of Pediatrics, School of Medicine; Department of Environmental and Occupational Health Sciences; School of Public Health	University of Washington	Seattle, Washington, USA	ECHO Cohort Study Site Principal Investigator

Supplemental Online Content: Nonauthor Collaborators
*First name, last name, and suffix (if applicable) are required and will appear in PubMed.

***First Name and Middle Initial(s)**	***Last Name**	***Suffix (eg, Jr, III)**	Academic Degrees	Institution	Location (city, state/province, country)	Role or Contribution, eg, chair, principal investigator	Group (if more than 1 Group listed in the byline) and/or Subgroup (eg, Steering Committee)
Nicole R.	Bush		PhD	Department of Psychiatry and Behavioral Sciences and Department of Pediatrics, School of Medicine	University of California, San Francisco	San Francisco, California, USA	ECHO Cohort Study Site Principal Investigator
Kaja Z.	LeWinn		ScD	Department of Psychiatry and Behavioral Sciences, School of Medicine	University of California, San Francisco	San Francisco, California, USA	ECHO Cohort Study Site Principal Investigator
Sheela	Sathyanarayana		MD, MPH	Department of Pediatrics, School of Medicine; Department of Environmental and Occupational Health Sciences, School of Public Health	University of Washington and Seattle Children's Research Institute	Seattle, Washington, USA	ECHO Cohort Study Site Principal Investigator
Qi	Zhao		MD, PhD	Department of Preventive Medicine	University of Tennessee Health Science Center	Memphis, Tennessee, USA	ECHO Cohort Study Site Principal Investigator
Frances	Tylavsky		DrPH, MS	Department of Preventive Medicine	University of Tennessee Health Science Center	Memphis, Tennessee, USA	ECHO Cohort Study Site Principal Investigator
Kecia N.	Carroll		MD, MPH	Department of Pediatrics, Department of Environmental Medicine & Public Health	Icahn School of Medicine at Mount Sinai	New York, New York, USA	ECHO Cohort Study Site Principal Investigator
Christine T.	Loftus		MS MPH PhD	Department of Environmental and Occupational Health Sciences; School of Public Health	University of Washington	Seattle, Washington, USA	ECHO Cohort Study Site Principal Investigator
Leslie D.	Leve		PhD	Department of Counseling Psychology and Human Services & Prevention Science Institute	University of Oregon	Eugene, Oregon, USA	ECHO Cohort Study Site Principal Investigator
Jody M.	Ganiban		PhD	Department of Psychological and Behavioral Sciences	George Washington University	Washington, DC, USA	ECHO Cohort Study Site Principal Investigator
Jenae M.	Neiderhiser		PhD	Department of Psychology	Penn State University	University Park, Pennsylvania, USA	ECHO Cohort Study Site Principal Investigator

*First name, last name, and suffix (if applicable) are required and will appear in PubMed.

***First Name and Middle Initial(s)**	***Last Name**	***Suffix (eg, Jr, III)**	Academic Degrees	Institution	Location (city, state/province, country)	Role or Contribution, eg, chair, principal investigator	Group (if more than 1 Group listed in the byline) and/or Subgroup (eg, Steering Committee)
Scott T.	Weiss		MD	Channing Division of Network Medicine, Department of Medicine	Brigham and Women's Hospital and Harvard Medical School	Boston, Massachusetts, USA	ECHO Cohort Study Site Principal Investigator
Augusto A.	Litonjua		MD	Pediatric Pulmonary Division, Department of Pediatrics	Golisano Children's Hospital, University of Rochester	Rochester, New York, USA	ECHO Cohort Study Site Principal Investigator
Cindy T.	McEvoy		MD, MCR	Division of Neonatology, Department of Pediatrics	Oregon Health & Science University	Portland, Oregon, USA	ECHO Cohort Study Site Principal Investigator
Eliot R.	Spindel		MD, PhD	Division of Neuroscience	Oregon National Primate Research Center	Beaverton, Oregon, USA	ECHO Cohort Study Site Principal Investigator
Robert S.	Tepper		MD, PhD	Division of Pediatric Pulmonology, Department of Pediatrics	Indiana School of Medicine	Indianapolis, Indiana, USA	ECHO Cohort Study Site Co-Investigator
Craig J.	Newschaffer		PhD	College of Health and Human Development	Penn State	State College, Pennsylvania, USA	ECHO Cohort Study Site Principal Investigator
Kristen	Lyall		ScD	AJ Drexel Autism Institute	Drexel University	Philadelphia, Pennsylvania, USA	ECHO Cohort Study Site Principal Investigator
Heather E.	Volk		PhD	Mental Health	Johns Hopkins University	Baltimore, Maryland, USA	ECHO Cohort Study Site Principal Investigator
Rebecca	Landa		PhD	Department of Psychiatry and Behavioral Sciences	Center for Autism and Related Disorders, Kennedy Krieger Institute, Johns Hopkins University	Baltimore, Maryland, USA	ECHO Cohort Study Site Co-Investigator
Sally	Ozonoff		PhD	MIND Institute, Department of Psychiatry	University of California Davis	Sacramento, California, USA	ECHO Cohort Study Site Co-Investigator
Joseph	Piven		MD	Department of Psychiatry	University of North Carolina	Chapel Hill, North Carolina, USA	ECHO Cohort Study Site Co-Investigator
Heather	Hazlett		PhD	Department of Psychiatry	University of North Carolina	Chapel Hill, North Carolina, USA	ECHO Cohort Study Site Co-Investigator
Juhi	Pandey		PhD	Center for Autism Research	Children's Hospital of Philadelphia	Philadelphia, Pennsylvania, USA	ECHO Cohort Study Site Co-Investigator

Supplemental Online Content: Nonauthor Collaborators
*First name, last name, and suffix (if applicable) are required and will appear in PubMed.

***First Name and Middle Initial(s)**	***Last Name**	***Suffix (eg, Jr, III)**	Academic Degrees	Institution	Location (city, state/province, country)	Role or Contribution, eg, chair, principal investigator	Group (if more than 1 Group listed in the byline) and/or Subgroup (eg, Steering Committee)
Robert	Schultz		PhD	Center for Autism Research	Children's Hospital of Philadelphia	Philadelphia, Pennsylvania, USA	ECHO Cohort Study Site Co-Investigator
Steven	Dager		PhD	Department of Radiology	University of Washington	Seattle, Washington, USA	ECHO Cohort Study Site Co-Investigator
Kelly	Botteron		PhD	Department of Psychiatry	Washington University	St Louis, Missouri, USA	ECHO Cohort Study Site Co-Investigator
Daniel	Messinger		PhD	Department of Psychology	University of Miami	Miami, Florida, USA	ECHO Cohort Study Site Co-Investigator
Wendy	Stone		PhD	Department of Psychology	University of Washington	Seattle, Washington, USA	ECHO Cohort Study Site Co-Investigator
Jennifer	Ames		PhD	Kaiser Permanente Division of Research	Kaiser Permanente	Oakland, California, USA	ECHO Cohort Study Site Co-Investigator
Thomas G.	O'Connor		PhD	Departments of Psychiatry, Neuroscience, Obstetrics and Gynecology	University of Rochester	Rochester, New York, USA	ECHO Cohort Study Site Principal Investigator
Richard K.	Miller		PhD	Departments of Obstetrics and Gynecology	University of Rochester	Rochester, New York, USA	ECHO Cohort Study Site Principal Investigator
Emily	Oken		MD, MPH	Division of Chronic Disease Research Across the Lifecourse, Department of Population Medicine	Harvard Pilgrim Health Care Institute and Harvard Medical School	Boston, Massachusetts, USA	ECHO Cohort Study Site Principal Investigator
Michele R.	Hacker		ScD	Department of Obstetrics and Gynecology	Beth Israel Deaconess Medical Center	Boston, Massachusetts, USA	ECHO Cohort Study Site Principal Investigator
Tamarra	James-Todd		PhD	Department of Environmental Health	Harvard Chan School of Public Health	Boston, Massachusetts, USA	ECHO Cohort Study Site Principal Investigator
T. Michael	O'Shea	Jr	MD, MPH	Division of Neonatology, Department of Pediatrics	University of North Carolina School of Medicine	Chapel Hill, North Carolina, USA	ECHO Cohort Study Site Principal Investigator
Rebecca C.	Fry		PhD	Department of Environmental Sciences and Engineering	University of North Carolina Gillings School of Global Public Health	Chapel Hill, North Carolina, USA	ECHO Cohort Study Site Principal Investigator

Supplemental Online Content: Nonauthor Collaborators
*First name, last name, and suffix (if applicable) are required and will appear in PubMed.

***First Name and Middle Initial(s)**	***Last Name**	***Suffix (eg, Jr, III)**	Academic Degrees	Institution	Location (city, state/province, country)	Role or Contribution, eg, chair, principal investigator	Group (if more than 1 Group listed in the byline) and/or Subgroup (eg, Steering Committee)
Jean A.	Frazier		MD	EK Shriver Center and Psychiatry	UMASS Chan Medical School	Worcster, Massachusetts, USA	ECHO Cohort Study Site Co-Investigator
Rachana	Singh		MD, MS	Department of Pediatrics	Tufts University School of Medicine	Boston, Massachusetts, USA	ECHO Cohort Study Site Co-Investigator
Caitlin	Rollins		MD, SM	Department of Neurology	Harvard Medical School	Boston, Massachusetts, USA	ECHO Cohort Study Site Co-Investigator
Angela	Montgomery		MD	Division of Neonatology, Department of Pediatrics	Yale School of Medicine	New Haven, Connecticut, USA	ECHO Cohort Study Site Co-Investigator
Ruben	Vaidya		MD	Department of Pediatrics	University of Massachusetts Chan Medical School-Baystate	Springfield, Massachusetts, USA	ECHO Cohort Study Site Co-Investigator
Robert M.	Joseph		PhD	Department of Anatomy & Neurobiology	Boston University Chobanian & Avedisian School of Medicine	Boston, Massachusetts, USA	ECHO Cohort Study Site Co-Investigator
Lisa K.	Washburn		MD	Pediatrics	Wake Forest School of Medicine	Winston-Salem, North Carolina, USA	ECHO Cohort Study Site Co-Investigator
Semsa	Gogcu		MD, MPH	Section of Neonatology, Department of Pediatrics; Department of Pediatrics	Wake Forest School of Medicine; Wake Forest University School of Medicine/Atrium Health Wake Forest	Winston-Salem, North Carolina, USA	ECHO Cohort Study Site Co-Investigator
Kelly	Bear		DO	Section of Neonatology, Department of Pediatrics	ECU Health	Greenville, North Carolina, USA	ECHO Cohort Study Site Co-Investigator
Julie V.	Rollins		MA	Division of Neonatology, Department of Pediatrics	University of North Carolina School of Medicine	Chapel Hill, North Carolina, USA	ECHO Cohort Study Site Award Project Director
Stephen R.	Hooper		PhD	Department of Health Sciences	School of Medicine, University of North Carolina at Chapel Hill	Chapel Hill, North Carolina, USA	ECHO Cohort Study Site Co-Investigator

Supplemental Online Content: Nonauthor Collaborators
*First name, last name, and suffix (if applicable) are required and will appear in PubMed.

***First Name and Middle Initial(s)**	***Last Name**	***Suffix (eg, Jr, III)**	Academic Degrees	Institution	Location (city, state/province, country)	Role or Contribution, eg, chair, principal investigator	Group (if more than 1 Group listed in the byline) and/or Subgroup (eg, Steering Committee)
Genevieve	Taylor		MD	Pediatrics	School of Medicine, University of North Carolina at Chapel Hill	Chapel Hill, North Carolina, USA	ECHO Cohort Study Site Co-Investigator
Wesley	Jackson		MD, MPH	Division of Neonatology, Department of Pediatrics	University of North Carolina School of Medicine	Chapel Hill, North Carolina, USA	ECHO Cohort Study Site Co-Investigator
Amanda	Thompson		PhD	Department of Anthropology, Department of Nutrition	University of North Carolina at Chapel Hill; Gillings School of Global Public Health, University of North Carolina at Chapel Hill	Chapel Hill, North Carolina, USA	ECHO Cohort Study Site Co-Investigator
Julie	Daniels		PhD	Epidemiology and Maternal and Child Health	University of North Carolina at Chapel Hill; Gillings School of Global Public Health, University of North Carolina at Chapel Hill	Chapel Hill, North Carolina, USA	ECHO Cohort Study Site Co-Investigator
Michelle	Hernandez		MD	Pediatrics	School of Medicine, University of North Carolina at Chapel Hill	Chapel Hill, North Carolina, USA	ECHO Cohort Study Site Co-Investigator
Kun	Lu		PhD	Environmental Sciences and Engineering	Gillings School of Global Public Health, University of North Carolina at Chapel Hill	Chapel Hill, North Carolina, USA	ECHO Cohort Study Site Co-Investigator
Michael	Msall		MD	Kennedy Research Center on Intellectual and Neurodevelopmental Disabilities	University of Chicago Medicine: Comer Children's Hospital	Chicago Illinois, USA	ECHO Cohort Study Site Co-Investigator
Madeleine	Lenski		MSPH	Department of Epidemiology and Biostatistics	Michigan State University	East Lansing, Michigan, USA	ECHO Cohort Study Site Co-Investigator

Supplemental Online Content: Nonauthor Collaborators
*First name, last name, and suffix (if applicable) are required and will appear in PubMed.

***First Name and Middle Initial(s)**	***Last Name**	***Suffix (eg, Jr, III)**	Academic Degrees	Institution	Location (city, state/province, country)	Role or Contribution, eg, chair, principal investigator	Group (if more than 1 Group listed in the byline) and/or Subgroup (eg, Steering Committee)
Rawad	Obeid		MD	Pediatrics	Beaumont Hospital	Royal Oak, Michigan, USA	ECHO Cohort Study Site Co-Investigator
Steven L.	Pastyrnak		PhD	Pediatrics	Corewell Health, Helen DeVos Children's Hospital	Grand Rapids, Michigan, USA	ECHO Cohort Study Site Co-Investigator
Elizabeth	Jensen		PhD	Epidemiology and Prevention	Wake Forest University School of Medicine	Winston-Salem, North Carolina, USA	ECHO Cohort Study Site Co-Investigator
Christina	Sakai		MD	Pediatrics	Mass General Hospital for Children	Boston, Massachusetts, USA	ECHO Cohort Study Site Co-Investigator
Hudson	Santos		RN, PhD	Dean's Office Graduate School, School of Nursing and Health Studies	University of Miami	Coral Gables, Florida, USA	ECHO Cohort Study Site Principal Investigator
Jean M.	Kerver		PhD, MSc, RD	Departments of Epidemiology & Biostatistics, and Pediatrics & Human Development	Michigan State University, College of Human Medicine	East Lansing, Michigan, USA	ECHO Cohort Study Site Principal Investigator
Nigel	Paneth		MD, MPH	Departments of Epidemiology & Biostatistics, and Pediatrics & Human Development	Michigan State University, College of Human Medicine	East Lansing, Michigan, USA	ECHO Cohort Study Site Principal Investigator
Charles J.	Barone	II	MD, FAAP	Department of Pediatrics	Henry Ford Health	Detroit, Michigan, USA	ECHO Cohort Study Site Principal Investigator
Michael R.	Elliott		PhD	Department of Biostatistics	University of Michigan	Ann Arbor, Michigan, USA	ECHO Cohort Study Site Principal Investigator
Douglas M.	Ruden		PhD	Department of Obstetrics and Gynecology, Institute of Environmental Health Sciences (IEHS), C.S. Mott Center for Human Health and Development	Wayne State University	Detroit, Michigan, USA	ECHO Cohort Study Site Principal Investigator
Chris	Fussman		MS	Lifecourse Epidemiology and Genomics Division	Michigan Department of Health and Human Services (MDHHS)	Lansing, Michigan, USA	ECHO Cohort Study Site Principal Investigator

Supplemental Online Content: Nonauthor Collaborators
*First name, last name, and suffix (if applicable) are required and will appear in PubMed.

***First Name and Middle Initial(s)**	***Last Name**	***Suffix (eg, Jr, III)**	Academic Degrees	Institution	Location (city, state/province, country)	Role or Contribution, eg, chair, principal investigator	Group (if more than 1 Group listed in the byline) and/or Subgroup (eg, Steering Committee)
Julie B.	Herbstman		PhD	Department of Environmental Health Sciences	Columbia University Mailman School of Public Health	New York, New York, USA	ECHO Cohort Study Site Principal Investigator
Amy	Margolis		PhD	Department of Psychiatry	Columbia University Irving Medical Center	New York, New York, USA	ECHO Cohort Study Site Principal Investigator
Susan L.	Schantz		PhD	Beckman Institute for Advanced Science and Technology; Department of Comparative Biosciences	University of Illinois Urbana-Champaign	Urbana, Illinois, USA	ECHO Cohort Study Site Principal Investigator
Sarah Dee	Geiger		PhD	Beckman Institute for Advanced Science and Technology; Department of Kinesiology and Community Health	University of Illinois Urbana-Champaign	Urbana, Illinois, USA	ECHO Cohort Study Site Co-Investigator
Andrea	Aguiar		PhD	Beckman Institute for Advanced Science and Technology; Department of Comparative Biosciences	University of Illinois Urbana-Champaign	Urbana, Illinois, USA	ECHO Cohort Study Site Co-Investigator
Karen	Tabb		PhD, MSW	Beckman Institute for Advanced Science and Technology; Department of Social Work	University of Illinois Urbana-Champaign	Urbana, Illinois, USA	ECHO Cohort Study Site Co-Investigator
Rita	Strakovsky		PhD	Department of Food Science and Human Nutrition	Michigan State University	East Lansing, Michigan, USA	ECHO Cohort Study Site Co-Investigator
Tracey	Woodruff		PhD, MPH	Program on Reproductive Health and the Environment	University of California, San Francisco	San Francisco, California, USA	ECHO Cohort Study Site Principal Investigator
Rachel	Morello-Frosch		PhD, MPH	Department of Environmental Science, Policy and Management and School of Public Health	University of California, Berkeley	Berkeley, California, USA	ECHO Cohort Study Site Principal Investigator
Amy	Padula		PhD	Program on Reproductive Health and the Environment	University of California, San Francisco	San Francisco, California, USA	ECHO Cohort Study Site Co-Investigator

Supplemental Online Content: Nonauthor Collaborators
*First name, last name, and suffix (if applicable) are required and will appear in PubMed.

***First Name and Middle Initial(s)**	***Last Name**	***Suffix (eg, Jr, III)**	Academic Degrees	Institution	Location (city, state/province, country)	Role or Contribution, eg, chair, principal investigator	Group (if more than 1 Group listed in the byline) and/or Subgroup (eg, Steering Committee)
Joseph B.	Stanford		MD, MSPH	Department of Family and Preventive Medicine	Spencer Fox Eccles School of Medicine, University of Utah	Salt Lake City, Utah, USA	ECHO Cohort Study Site Principal Investigator
Christina A.	Porucznik		PhD, MSPH	Department of Family and Preventive Medicine	Spencer Fox Eccles School of Medicine, University of Utah	Salt Lake City, Utah, USA	ECHO Cohort Study Site Principal Investigator
Angelo P.	Giardino		MD, PhD	Department of Pediatrics	Spencer Fox Eccles School of Medicine, University of Utah	Salt Lake City, Utah, USA	ECHO Cohort Study Site Principal Investigator
Rosalind J.	Wright		MD, MPH	Department of Environmental Medicine & Public Health	Icahn School of Medicine at Mount Sinai	New York, New York, USA	ECHO Cohort Study Site Principal Investigator
Robert O.	Wright		MD, MPH	Department of Environmental Medicine & Public Health	Icahn School of Medicine at Mount Sinai	New York, New York, USA	ECHO Cohort Study Site Principal Investigator
Brent	Collett		PhD	Department of Psychiatry and Behavioral Medicine	University of Washington, Seattle Children's Research Institute	Seattle, Washington, USA	ECHO Cohort Study Site Principal Investigator
Nicole	Baumann-Blackmore		MD	Department of Pediatrics	University of Wisconsin School of Medicine and Public Health	Madison, Wisconsin, USA	ECHO Cohort Study Site Co-Investigator
Ronald	Gangnon		PhD	Department of Population Health Sciences	University of Wisconsin	Madison, Wisconsin, USA	ECHO Cohort Study Site Co-Investigator
Daniel J.	Jackson		MD	Department of Pediatrics	University of Wisconsin School of Medicine and Public Health	Madison, Wisconsin, USA	ECHO Cohort Study Site Co-Investigator
Chris G.	McKennan		PhD	Department of Statistics	University of Pittsburgh	Pittsburgh, Pennsylvania, USA	ECHO Cohort Study Site Co-Investigator
Jo	Wilson		MD	Department of Pediatrics	University of Wisconsin School of Medicine and Public Health	Madison, Wisconsin, USA	ECHO Cohort Study Site Co-Investigator

Supplemental Online Content: Nonauthor Collaborators
*First name, last name, and suffix (if applicable) are required and will appear in PubMed.

***First Name and Middle Initial(s)**	***Last Name**	***Suffix (eg, Jr, III)**	Academic Degrees	Institution	Location (city, state/province, country)	Role or Contribution, eg, chair, principal investigator	Group (if more than 1 Group listed in the byline) and/or Subgroup (eg, Steering Committee)
Matt	Altman		MD	Department of Medicine	University of Washington	Seattle, Washington, USA	ECHO Cohort Study Site Co-Investigator
Judy L.	Aschner		MD	Department of Pediatrics	Albert Einstein College of Medicine; Hackensack Meridian School of Medicine; Center for Discovery and Innovation	Bronx, New York, USA; Nutley, New Jersey, USA	ECHO Cohort Study Site Principal Investigator
Annemarie	Stroustrup		MD, MPH	Department of Pediatrics	Northwell Health, Cohen Children's Medical Center, and the Zucker School of Medicine at Hofstra / Northwell	New Hyde Park, New York, USA	ECHO Cohort Study Site Principal Investigator
Stephanie L.	Merhar		MD, MS	Department of Pediatrics	Cincinnati Children's	Cincinnati, Ohio, USA	ECHO Cohort Study Site Co-Investigator
Paul E.	Moore		MD	Department of Pediatrics	Vanderbilt University Medical Center	Nashville, Tennessee, USA	ECHO Cohort Study Site Co-Investigator
Gloria S.	Pryhuber		MD	Department of Pediatrics	University of Rochester Medical Center	Rochester, New York, USA	ECHO Cohort Study Site Co-Investigator
Mark	Hudak		MD	Department of Pediatrics	University of Florida College of Medicine	Jacksonville, Florida, USA	ECHO Cohort Study Site Co-Investigator
Ann Marie	Reynolds Lyndaker		MD, MPH	Department of Pediatrics	University of Buffalo Jacobs School of Medicine and Biomedical Sciences	Buffalo, New York, USA	ECHO Cohort Study Site Co-Investigator
Andrea L.	Lampland		MD	Department of Pediatrics	Children's Minnesota	Minneapolis, Minnesota, USA	ECHO Cohort Study Site Co-Investigator
Burton	Rochelson		MD	Department of Obstetrics and Gynecology	Northwell Health and the Zucker School of Medicine at Hofstra / Northwell	New Hyde Park, New York, USA	ECHO Cohort Study Site Principal Investigator

Supplemental Online Content: Nonauthor Collaborators
*First name, last name, and suffix (if applicable) are required and will appear in PubMed.

***First Name and Middle Initial(s)**	***Last Name**	***Suffix (eg, Jr, III)**	Academic Degrees	Institution	Location (city, state/province, country)	Role or Contribution, eg, chair, principal investigator	Group (if more than 1 Group listed in the byline) and/or Subgroup (eg, Steering Committee)
Sophia	Jan		MD, MSHP	Department of Pediatrics	Northwell Health, Cohen Children's Medical Center, and the Zucker School of Medicine at Hofstra / Northwell	New Hyde Park, New York, USA	ECHO Cohort Study Site Co-Investigator
Matthew J.	Blitz		MD, MBA	Department of Obstetrics and Gynecology	Northwell Health and the Zucker School of Medicine at Hofstra / Northwell	New Hyde Park, New York, USA	ECHO Cohort Study Site Co-Investigator
Michelle W.	Katzow		MD, MS	Department of Pediatrics	Northwell Health, Cohen Children's Medical Center, and the Zucker School of Medicine at Hofstra / Northwell	New Hyde Park, New York, USA	ECHO Cohort Study Site Co-Investigator
Zenobia	Brown		MD, MPH	Department of Science Education	Northwell Health and the Zucker School of Medicine at Hofstra / Northwell	New Hyde Park, New York, USA	ECHO Cohort Study Site Co-Investigator
Codruta	Chiuzan		PhD	Institute of Health System Science	Northwell Health, Feinstein Institutes for Medical Research	Manhasset, New York, USA	ECHO Cohort Study Site Co-Investigator
Timothy	Rafael		MD	Department of Obstetrics and Gynecology	Northwell Health and the Zucker School of Medicine at Hofstra / Northwell	New Hyde Park, New York, USA	ECHO Cohort Study Site Co-Investigator
Dawnette	Lewis		MD, MPH	Department of Obstetrics and Gynecology	Northwell Health and the Zucker School of Medicine at Hofstra / Northwell	New Hyde Park, New York, USA	ECHO Cohort Study Site Co-Investigator

Supplemental Online Content: Nonauthor Collaborators
*First name, last name, and suffix (if applicable) are required and will appear in PubMed.

***First Name and Middle Initial(s)**	***Last Name**	***Suffix (eg, Jr, III)**	Academic Degrees	Institution	Location (city, state/province, country)	Role or Contribution, eg, chair, principal investigator	Group (if more than 1 Group listed in the byline) and/or Subgroup (eg, Steering Committee)
Natalie	Meirowitz		MD	Department of Obstetrics and Gynecology	Northwell Health and the Zucker School of Medicine at Hofstra / Northwell	New Hyde Park, New York, USA	ECHO Cohort Study Site Co-Investigator
Brenda	Poindexter		MD	Department of Pediatrics	Children's Healthcare of Atlanta Emory University	Atlanta, Georgia, USA	ECHO Cohort Study Site Co-Investigator
Tebeb	Gebretsadik		MPH	Department of Biostatistics	Vanderbilt University Medical Center	Nashville, Tennessee, USA	ECHO Cohort Study Site Principal Investigator
Sarah	Osmundson		MD, MSC	Department of Obstetrics and Gynecology	Vanderbilt University Medical Center	Nashville, Tennessee, USA	ECHO Cohort Study Site Principal Investigator
Jennifer K.	Straughen		PhD	Department of Public Health Sciences	Henry Ford Health	Detroit, Michigan, USA	ECHO Cohort Study Site Principal Investigator
Amy	Eapen		MD	Division of Allergy and Clinical Immunology	Henry Ford Health	Detroit, Michigan, USA	ECHO Cohort Study Site Principal Investigator
Andrea	Cassidy-Bushrow		PhD	Department of Public Health Sciences	Henry Ford Health	Detroit, Michigan, USA	ECHO Cohort Study Site Co-Investigator
Ganesa	Wegienka		PhD	Department of Public Health Sciences	Henry Ford Health	Detroit, Michigan, USA	ECHO Cohort Study Site Co-Investigator
Alex	Sitarik		MPH	Department of Public Health Sciences	Henry Ford Health	Detroit, Michigan, USA	ECHO Cohort Study Site Biostatistician
Kim	Woodcroft		PhD	Department of Public Health Sciences	Henry Ford Health	Detroit, Michigan, USA	ECHO Cohort Study Site Co-Investigator
Audrey	Urquhart		MPH	Department of Public Health Sciences	Henry Ford Health	Detroit, Michigan, USA	ECHO Cohort Study Site Epidemiologist
Albert	Levin		PhD	Department of Public Health Sciences	Henry Ford Health	Detroit, Michigan, USA	ECHO Cohort Study Site Co-Investigator
Tisa	Johnson-Hooper		MD	Department of Pediatrics	Henry Ford Health	Detroit, Michigan, USA	ECHO Cohort Study Site Co-Investigator
Brent	Davidson		MD	Department of Women's Health	Henry Ford Health	Detroit, Michigan, USA	ECHO Cohort Study Site Co-Investigator

*First name, last name, and suffix (if applicable) are required and will appear in PubMed.

***First Name and Middle Initial(s)**	***Last Name**	***Suffix (eg, Jr, III)**	Academic Degrees	Institution	Location (city, state/province, country)	Role or Contribution, eg, chair, principal investigator	Group (if more than 1 Group listed in the byline) and/or Subgroup (eg, Steering Committee)
Tengfei	Ma		PhD	Department of Public Health Sciences	Henry Ford Health	Detroit, Michigan, USA	ECHO Cohort Study Site Co-Investigator
Emily S.	Barrett		PhD	Department of Biostatistics and Epidemiology	Environmental and Occupational Health Sciences Institute, Rutgers University	Piscataway, New Jersey, USA	ECHO Cohort Study Site Principal Investigator
Martin J.	Blaser		MD	Center for Advanced Biotechnology & Medicine	Rutgers University	Piscataway, New Jersey, USA	ECHO Cohort Study Site Principal Investigator
Maria Gloria	Dominguez-Bello		PhD	Departments of Biochemistry and Microbiology & Anthropology	Rutgers University	New Brunswick, New Jersey, USA	ECHO Cohort Study Site Principal Investigator
Daniel B.	Horton		MD	Department of Pediatrics	Robert Wood Johnson Medical School, Rutgers University	New Brunswick, New Jersey, USA	ECHO Cohort Study Site Principal Investigator
Manuel	Jimenez		MD	Departments of Pediatrics, Family Medicine, and Community Health	Robert Wood Johnson Medical School, Rutgers University	New Brunswick, New Jersey, USA	ECHO Cohort Study Site Principal Investigator
Todd	Rosen		MD	Department of Obstetrics, Gynecology, and Reproductive Sciences	Robert Wood Johnson Medical School, Rutgers University	New Brunswick, New Jersey, USA	ECHO Cohort Study Site Co-Investigator
Kristy	Palomares		MD, PhD	Department of Obstetrics and Gynecology	Saint Peter's University Hospital	New Brunswick, New Jersey, USA	ECHO Cohort Study Site Co-Investigator
Lyndsay A.	Avalos		PhD, MPH	Division of Research	Kaiser Permanente Northern California	Oakland, California, USA	ECHO Cohort Study Site Principal Investigator
Yeyi	Zhu		PhD, MS	Division of Research	Kaiser Permanente Northern California	Oakland, California, USA	ECHO Cohort Study Site Principal Investigator
Kelly J .	Hunt		PhD	Department of Public Health Sciences	Medical University of South Carolina	Charleston, South Carolina, USA	ECHO Cohort Study Site Principal Investigator
Roger B.	Newman		MD	Department of Obstetrics and Gynecology	Medical University of South Carolina	Charleston, South Carolina, USA	ECHO Cohort Study Site Principal Investigator
Michael S.	Bloom		PhD	Department of Global and Community Health	George Mason University	Fairfax, Virginia, USA	ECHO Cohort Study Site Principal Investigator

Supplemental Online Content: Nonauthor Collaborators
*First name, last name, and suffix (if applicable) are required and will appear in PubMed.

***First Name and Middle Initial(s)**	***Last Name**	***Suffix (eg, Jr, III)**	Academic Degrees	Institution	Location (city, state/province, country)	Role or Contribution, eg, chair, principal investigator	Group (if more than 1 Group listed in the byline) and/or Subgroup (eg, Steering Committee)
Mallory H.	Alkis		MD	Department of Obstetrics and Gynecology	Medical University of South Carolina	Charleston, South Carolina, USA	ECHO Cohort Study Site Co-Investigator
James R.	Roberts		MD, MPH	Department of Pediatrics	Medical University of South Carolina	Charleston, South Carolina, USA	ECHO Cohort Study Site Co-Investigator
Sunni L.	Mumford		PhD	Department of Biostatistics, Epidemiology and Informatics; Department of Obstetrics and Gynecology	University of Pennsylvania Perelman School of Medicine	Philadelphia, Pennsylvania, USA	ECHO Cohort Study Site Principal Investigator
Heather H.	Burris		MD, MPH	Division of Neonatology, Department of Pediatrics	Children's Hospital of Philadelphia; University of Pennsylvania Perelman School of Medicine	Philadelphia, Pennsylvania, USA	ECHO Cohort Study Site Principal Investigator
Sara B.	DeMauro		MD, MSCE	Division of Neonatology, Department of Pediatrics	Children's Hospital of Philadelphia; University of Pennsylvania Perelman School of Medicine	Philadelphia, Pennsylvania, USA	ECHO Cohort Study Site Principal Investigator
Lynn M.	Yee		MD, MPH	Division of Maternal-Fetal Medicine, Department of Obstetrics & Gynecology	Feinberg School of Medicine, Northwestern University	Chicago, Illinois, USA	ECHO Cohort Study Site Principal Investigator
Aaron	Hamvas		MD	Division of Neonatology, Department of Pediatrics	Ann & Robert H. Lurie Children's Hospital, Feinberg School of Medicine, Northwestern University	Chicago, Illinois, USA	ECHO Cohort Study Site Principal Investigator
Antonia F.	Olidipo		MD, MSCI	Division of Maternal-Fetal Medicine, Department of Obstetrics & Gynecology	Hackensack University Medical Center, Hackensack Meridian School of Medicine	Nutley, New Jersey, USA	ECHO Cohort Study Site Co-Investigator

Supplemental Online Content: Nonauthor Collaborators
*First name, last name, and suffix (if applicable) are required and will appear in PubMed.

***First Name and Middle Initial(s)**	***Last Name**	***Suffix (eg, Jr, III)**	Academic Degrees	Institution	Location (city, state/province, country)	Role or Contribution, eg, chair, principal investigator	Group (if more than 1 Group listed in the byline) and/or Subgroup (eg, Steering Committee)
Andrew S.	Haddad		MD	Division of Maternal-Fetal Medicine, Department of Obstetrics & Gynecology	Hackensack University Medical Center, Hackensack Meridian School of Medicine	Nutley, New Jersey, USA	ECHO Cohort Study Site Co-Investigator
Lisa R.	Eiland		MD	Division of Neonatology, Department of Pediatrics	Hackensack University Medical Center, Hackensack Meridian School of Medicine	Nutley, New Jersey, USA	ECHO Cohort Study Site Co-Investigator
Nicole T.	Spillane		MD	Division of Neonatology, Department of Pediatrics	Hackensack University Medical Center, Hackensack Meridian School of Medicine	Nutley, New Jersey, USA	ECHO Cohort Study Site Co-Investigator
Kirin N.	Suri		MD	Division of Developmental and Behavioral Pediatrics, Department of Pediatrics	Hackensack University Medical Center, Hackensack Meridian School of Medicine	Nutley, New Jersey, USA	ECHO Cohort Study Site Co-Investigator
Stephanie A.	Fisher		MD, MPH	Division of Maternal-Fetal Medicine, Department of Obstetrics & Gynecology	Feinberg School of Medicine, Northwestern University	Chicago, Illinois, USA	ECHO Cohort Study Site Co-Investigator
Jeffrey A.	Goldstein		MD, PhD	Department of Pathology	Feinberg School of Medicine, Northwestern University	Chicago, Illinois, USA	ECHO Cohort Study Site Co-Investigator
Leena B.	Mithal		MD	Division of Infectious Diseases, Department of Pediatrics	Ann & Robert H. Lurie Children's Hospital, Feinberg School of Medicine, Northwestern University	Chicago, Illinois, USA	ECHO Cohort Study Site Co-Investigator

Supplemental Online Content: Nonauthor Collaborators
*First name, last name, and suffix (if applicable) are required and will appear in PubMed.

***First Name and Middle Initial(s)**	***Last Name**	***Suffix (eg, Jr, III)**	Academic Degrees	Institution	Location (city, state/province, country)	Role or Contribution, eg, chair, principal investigator	Group (if more than 1 Group listed in the byline) and/or Subgroup (eg, Steering Committee)
Raye-Ann O.	DeRegnier		MD	Division of Neonatology, Department of Pediatrics	Ann & Robert H. Lurie Children's Hospital, Feinberg School of Medicine, Northwestern University	Chicago, Illinois, USA	ECHO Cohort Study Site Co-Investigator
Nathalie L.	Maitre		MD, PhD	Division of Neonatology, Department of Pediatrics	Emory University School of Medicine and Cerebral Palsy Foundation	Atlanta, Georgia, USA and New York, New York, USA	ECHO Cohort Study Site Co-Investigator
Ruby H.N.	Nguyen		PhD, MHS	Division of Epidemiology & Community Health	School of Public Health, University of Minnesota	Minneapolis, Minnesota, USA	ECHO award Principal Investigator
Meghan M.	JaKa		PhD, MS	Division of Research & Evaluation	HealthPartners Institute	Minneapolis, Minnesota, USA	ECHO site Principal Investigator
Abbey C.	Sidebottom		PhD, MPH	Care Delivery Research	Allina Health	Minneapolis, Minnesota, USA	ECHO site Principal Investigator
Michael J.	Paidas		MD	Department of Obstetrics and Gynecology	University of Miami Miller School of Medicine	Miami, Florida, USA	ECHO site Principal Investigator
JoNell E.	Potter		APRN, PhD	Department of Obstetrics, Gynecology and Reproductive Sciences	University of Miami Miller School of Medicine	Miami, Florida, USA	ECHO Cohort Study Site Co-Investigator
Natale	Ruby		PhD, PsyD	Mailman Center for Child Development	University of Miami Miller School of Medicine	Miami, Florida, USA	ECHO Cohort Study Site Co-Investigator
Lunthita	Duthely		EdD	Department of Obstetrics, Gynecology and Reproductive Sciences and Department of Public Health Sciences	University of Miami School of Medicine	Miami, Florida, USA	ECHO Cohort Study Site Co-Investigator
Arumugam	Jayakumar		PhD	Department of Obstetrics, Gynecology and Reproductive Sciences	University of Miami Miller School of Medicine	Miami, Florida, USA	ECHO Cohort Study Site Co-Investigator

Supplemental Online Content: Nonauthor Collaborators
*First name, last name, and suffix (if applicable) are required and will appear in PubMed.

***First Name and Middle Initial(s)**	***Last Name**	***Suffix (eg, Jr, III)**	Academic Degrees	Institution	Location (city, state/province, country)	Role or Contribution, eg, chair, principal investigator	Group (if more than 1 Group listed in the byline) and/or Subgroup (eg, Steering Committee)
Karen	Young		MD	Department of Pediatrics	University of Miami Miller School of Medicine	Miami, Florida, USA	ECHO Cohort Study Site Co-Investigator
Isabel	Maldonado		MPH, BS	School of Nursing and Health Studies	University of Miami	Miami, Florida, USA	ECHO Cohort Study Site Program Director
Meghan	Miller		PhD	Psychiatry and Behavioral Sciences; MIND Institute	University of California Davis	Sacramento, California, USA	ECHO Cohort Study Site Co-Investigator
Jonathan L.	Slaughter		MD, MPH	Center for Perinatal Research, Abigail Wexner Research Institute and Division of Neonatology, Nationwide Children's Hospital and Department of Pediatrics, College of Medicine and Division of Epidemiology, College of Public Health, The Ohio State University	Nationwide Children's Hospital and The Ohio State University	Columbus, Ohio, USA	ECHO Cohort Study Site Principal Investigator
Sarah A.	Keim		PhD, MS, MA	Center for Biobehavioral Health, Abigail Wexner Research Institute, Nationwide Children's Hospital and Department of Pediatrics, College of Medicine and Division of Epidemiology, College of Public Health, The Ohio State University	Nationwide Children's Hospital and The Ohio State University	Columbus, Ohio, USA	ECHO Cohort Study Site Principal Investigator
Courtney D.	Lynch		PhD, MPH	Division of Maternal-Fetal Medicine, Department of Obstetrics and Gynecology, College of Medicine and Division of Epidemiology, College of Public Health, The Ohio State University	The Ohio State University	Columbus, Ohio, USA	ECHO Cohort Study Site Principal Investigator

Supplemental Online Content: Nonauthor Collaborators
*First name, last name, and suffix (if applicable) are required and will appear in PubMed.

***First Name and Middle Initial(s)**	***Last Name**	***Suffix (eg, Jr, III)**	Academic Degrees	Institution	Location (city, state/province, country)	Role or Contribution, eg, chair, principal investigator	Group (if more than 1 Group listed in the byline) and/or Subgroup (eg, Steering Committee)
Kartik K.	Venkatesh		MD, PhD	Division of Maternal-Fetal Medicine, Department of Obstetrics and Gynecology, College of Medicine and Division of Epidemiology, College of Public Health, The Ohio State University	The Ohio State University	Columbus, Ohio, USA	ECHO Cohort Study Site Principal Investigator
Kristina W.	Whitworth		PhD	Center for Precision Environmental Health and Department of Medicine	Baylor College of Medicine	Houston, Texas, USA	ECHO Cohort Study Site Principal Investigator
Elaine	Symanski		PhD	Center for Precision Environmental Health and Department of Medicine	Baylor College of Medicine	Houston, Texas, USA	ECHO Cohort Study Site Principal Investigator
Thomas F.	Northrup		PhD	Department of Family and Community Medicine	University of Texas Health Science Center at Houston (UTHealth Houston) McGovern Medical School	Houston, Texas, USA	ECHO Cohort Study Site Principal Investigator
Hector	Mendez-Figueroa		MD	Department of Obstetrics, Gynecology and Reproductive Sciences	University of Texas Health Science Center at Houston (UTHealth Houston) McGovern Medical School	Houston, Texas, USA	ECHO Cohort Study Site Co-Investigator
Ricardo A.	Mosquera		MD	Department of Pediatrics	University of Texas Health Science Center at Houston (UTHealth Houston) McGovern Medical School	Houston, Texas, USA	ECHO Cohort Study Site Co-Investigator
Margaret R.	Karagas		PhD	Department of Epidemiology	Geisel School of Medicine at Dartmouth	Hanover, New Hampshire, USA	ECHO Cohort Study Site Principal Investigator

Supplemental Online Content: Nonauthor Collaborators
*First name, last name, and suffix (if applicable) are required and will appear in PubMed.

***First Name and Middle Initial(s)**	***Last Name**	***Suffix (eg, Jr, III)**	Academic Degrees	Institution	Location (city, state/province, country)	Role or Contribution, eg, chair, principal investigator	Group (if more than 1 Group listed in the byline) and/or Subgroup (eg, Steering Committee)
Juliette C.	Madan		MD, MS	Departments of Psychiatry, Pediatrics & Epidemiology	Geisel School of Medicine at Dartmouth, Dartmouth Hitchcock Medical Center	Hanover, New Hampshire, USA	ECHO Cohort Study Site Principal Investigator
Debra M.	MacKenzie		PhD	Community Environmental Health Program, Department of Pharmaceutical Sciences	College of Pharmacy, University of New Mexico Health Sciences Center	Albuquerque, New Mexico, USA	ECHO Cohort Study Site Principal Investigator
Johnnye L.	Lewis		PhD	Community Environmental Health Program, Department of Pharmaceutical Sciences	College of Pharmacy, University of New Mexico Health Sciences Center	Albuquerque, New Mexico, USA	ECHO Cohort Study Site Principal Investigator
Brandon J.	Rennie		PhD	Center for Development and Disability	University of New Mexico	Albuquerque, New Mexico, USA	ECHO Cohort Study Site Co-Investigator
Bennett L.	Leventhal		MD	Community Environmental Health Program, Department of Pharmaceutical Sciences UNM	College of Pharmacy, University of New Mexico Health Sciences Center; University of Chicago	Albuquerque, New Mexico, USA; Chicago, Illinois, USA	ECHO Cohort Study Site Co-Investigator
Young Shin	Kim		MD, MS, MPH, PhD	Department of Psychiatry and Behavioral Sciences	University of California, San Francisco	San Francisco, California, USA	ECHO Cohort Study Site Co-Investigator
Somer	Bishop		PhD	Department of Psychiatry and Behavioral Sciences	University of California, San Francisco	San Francisco, California, USA	ECHO Cohort Study Site Co-Investigator
Sara S.	Nozadi		PhD	Community Environmental Health Program, Department of Pharmaceutical Sciences	College of Pharmacy, University of New Mexico Health Sciences Center	Albuquerque, New Mexico, USA	ECHO Cohort Study Site Co-Investigator
Li	Luo		PhD	Department of Internal Medicine	Comprehensive Cancer Center, University of New Mexico Health Sciences Center	Albuquerque, New Mexico, USA	ECHO Cohort Study Site Co-Investigator

*First name, last name, and suffix (if applicable) are required and will appear in PubMed.

***First Name and Middle Initial(s)**	***Last Name**	***Suffix (eg, Jr, III)**	Academic Degrees	Institution	Location (city, state/province, country)	Role or Contribution, eg, chair, principal investigator	Group (if more than 1 Group listed in the byline) and/or Subgroup (eg, Steering Committee)
Barry M.	Lester		PhD	Department of Pediatrics, Department of Psychiatry and Human Behavior	Warren Alpert Medical School of Brown University	Providence, Rhode Island, USA	ECHO Cohort Study Site Principal Investigator
Carmen J.	Marsit		PhD	Department of Environmental Health	Rollins School of Public Health, Emory University	Atlanta, Georgia, USA	ECHO Cohort Study Site Principal Investigator
Todd	Everson		PhD	Department of Environmental Health	Rollins School of Public Health, Emory University	Atlanta, Georgia, USA	ECHO Cohort Study Site Principal Investigator
Cynthia M.	Loncar		PhD	Department of Psychiatry and Human Behavior	Warren Alpert Medical School of Brown University	Providence, Rhode Island, USA	ECHO Cohort Study Site Principal Investigator
Elisabeth C.	McGowan		MD	Department of Pediatrics	Warren Alpert Medical School of Brown University	Providence, Rhode Island, USA	ECHO Cohort Study Site Principal Investigator
Stephen J.	Sheinkopf		PhD	Department of Pediatrics	Thompson Center for Autism & Neurodevelopment, University of Missouri	Columbia, Missouri, USA	ECHO Cohort Study Site Principal Investigator
Brian S.	Carter		MD	Department of Pediatrics	Children's Mercy-Kansas City	Kansas City, Missouri, USA	ECHO Cohort Study Site Principal Investigator
Jennifer	Check		MD	Department of Pediatrics	Wake Forest School of Medicine	Winston, Salem North Carolina, USA	ECHO Cohort Study Site Principal Investigator
Jennifer B.	Helderman		MD	Department of Pediatrics	Wake Forest School of Medicine	Winston, Salem North Carolina, USA	ECHO Cohort Study Site Principal Investigator
Charles R.	Neal		MD	Department of Pediatrics	University of Hawaii John A Burns School of Medicine	Honolulu, Hawaii, USA	ECHO Cohort Study Site Principal Investigator

Supplemental Online Content: Nonauthor Collaborators
*First name, last name, and suffix (if applicable) are required and will appear in PubMed.

***First Name and Middle Initial(s)**	***Last Name**	***Suffix (eg, Jr, III)**	Academic Degrees	Institution	Location (city, state/province, country)	Role or Contribution, eg, chair, principal investigator	Group (if more than 1 Group listed in the byline) and/or Subgroup (eg, Steering Committee)
Lynne M.	Smith		MD	Department of Pediatrics	UCLA Clinical and Translational Science Institute at The Lundquist Institute, Harbor-UCLA Medical Center	Los Angeles, California, USA	ECHO Cohort Study Site Principal Investigator
