# Supplementary material for: Subfecundity, Infertility Treatment, and Child Neurodevelopment
Source: JAMA Netw Open. 2026 Jun 8;9(6):e2617324. doi: 10.1001/jamanetworkopen.2026.17324 (PMC13247815; doi:10.1001/jamanetworkopen.2026.17324)
Supplement: Supplement 3. — Data Sharing Statement [file jamanetwopen-e2617324-s003.pdf]

Data Sharing Statement

Kahn. Subfecundity, Infertility Treatment, and Child Neurodevelopment. *JAMA Netw Open*. Published June 08, 2026. doi:10.1001/jamanetworkopen.2026.17324

Data
Data available: Yes
Data types: Deidentified participant data
How to access data: ECHO Cohort data may be accessed via the NICHD Data and Specimen Hub (DASH): https://dash.nichd.nih.gov/
When available: With publication

Supporting Documents
Document types: None

Additional Information
Who can access the data: Data will be made available to researchers whose proposals are reviewed and approved by NICHD DASH.
Types of analyses: Data will be made available for studies that align with the aim of the ECHO Cohort: to understand the effects of environmental influences on child health outcomes.
Mechanisms of data availability: Data will be made available following the approval of a research proposal submitted to NICHD DASH.
